# Human placental mesenchymal stem cells improve stroke outcomes via extracellular vesicles-mediated preservation of cerebral blood flow

**DOI:** 10.1016/j.ebiom.2020.103161

**Published:** 2020-12-19

**Authors:** Mansoureh Barzegar, Yuping Wang, Randa S. Eshaq, J. Winny Yun, Christen J. Boyer, Sergio G. Cananzi, Luke A. White, Oleg Chernyshev, Roger E. Kelley, Alireza Minagar, Karen Y. Stokes, Xiao-Hong Lu, Jonathan S. Alexander

**Affiliations:** a Molecular and Cellular Physiology, Medicine and Neurology, Ochsner-LSU Health Sciences Center-1501 Kings Highway, Shreveport, LA 71130-3932, USA; b Obstetrics and Gynecology and Medicine, Ochsner-LSU Health Sciences Center-Shreveport, LA 71130, USA; c Molecular Biology, UT Southwestern Medical Center, Dallas, TX 75390, USA; d Neurology, Ochsner-LSU Health Sciences Center-Shreveport, LA 71130, USA; e Pharmacology and Neuroscience, Ochsner-LSU Health Sciences Center-Shreveport, LA 71130, USA

**Keywords:** Ischemic stroke, Human placental mesenchymal stem cells, Extracellular vesicles, Cerebral blood flow, Infarction, Blood brain barrier

## Abstract

**Background:**

Besides long-term trans-differentiation into neural cells, benefits of stem cell therapy (SCT) in ischemic stroke may include secretion of protective factors, which partly reflects extracellular vesicle (EVs) released by stem cell. However, the mechanism(s) by which stem cells/EVs limit stroke injury have yet to be fully defined.

**Methods:**

We evaluated the protection effect of human placenta mesenchymal stem cells (hPMSC) as a potential form of SCT in experimental ischemic stroke ‘transient middle cerebral artery occusion (MCAO)/reperfusion’ mice model.

**Findings:**

We found for the first time that intraperitoneal administration of hPMSCs or intravenous hPMSC-derived EVs, given at the time of reperfusion, significantly protected the ipsilateral hemisphere from ischemic injury. This protection was associated with significant restoration of normal blood flow to the post-MCAO brain. More importantly, EVs derived from hPMSC promote paracrine-based protection of SCT in the MCAO model in a cholesterol/lipid-dependent manner.

**Interpretation:**

Together, our results demonstrated beneficial effects of hPMSC/EVs in experimental stroke models which could permit the rapid “translation” of these cells into clinical trials in the near-term.

**Research in context**

**Evidence before this study**

Although stem cell therapy for stroke has been previously studied by several groups and in several models, stem cells have still not yet been widely implemented as a therapy in acute stroke for several reasons. The failure for stem cells to be used in stroke treatment may represent in large part the significant risk for development of intravascular thrombi when given intravenously in clinical states. To our knowledge few, if any, studies have so far considered whether or to what extent intraperitoneal administration of stem cells might also provide potent acute stroke protection.

**Added value of this study**

The conceptual advance provided by our current study is to show that intraperitoneal administration of human placenta mesenchymal stem cells (hPMSC) in MCAO model are powerfully protective against acute stroke injury. Strikingly, these benefits are consistent with *paracrine functions of extracellular vesicles* derived from hPMSC as biochemical manipulation of membrane cholesterol can positively and negatively alter this protective effect. Our finding also demonstrated that how hPMSC might be safely used in acute therapy for ischemic stroke as this novel approach (intraperitoneal injection of hPMSC) is therapeutically far superior to intravenous stem cell therapy in terms of efficacy.

**Implications of all the available evidence**

Because this work describes an important and novel set of properties of hPMSC and their derivatives in stem cell therapy; their applications could be rapidly translated as a promising approach for treating the acute-post ischemic phase in human stroke therapy.

Alt-text: Unlabelled box

## 1 Introduction

In the US, stroke remains the leading cause of neurologically-mediated disability, and the 3rd leading cause of mortality in adults [1] with stroke incidence and occurrence increasing proportionately with aging in both developed and developing nations. A thromboembolic/ischemic mechanism accounts for up to 85% of stroke with up to 15% hemorrhagic [2]. Ischemic strokes reflect an acute and progressive destruction of neurons, astroglia and oligodendroglia with disruption of the cortical synaptic structure [1,3]. Maintenance of cerebral blood flow (CBF) is critical for brain function [4] with several protective auto-regulatory mechanisms which ensure adequate perfusion to cerebral arteries under variable conditions. Because of the large cerebral energy demand, it is critical to optimally restore CBF in the acute phase of stroke. A treatment that has been demonstrated to reduce brain damage after stroke is tissue plasminogen activator (t-PA), an enzyme which converts plasminogen to plasmin that dissolves emboli and thrombi [5], thereby restoring CBF. However, tPA is primarily effective in stroke if administered within 4–5 h of the onset of ischemia. Paradoxically, the act of restoring local blood perfusion can triggers ischemia/reperfusion injury (IRI) that intensifies stroke severity. Several events contribute to IRI including depletion of energy and oxygen supply, inflammatory infiltration of neutrophils and macrophages into brain tissue [6,7], impairment of the blood brain barrier (BBB) [2,8] and disturbed vasoregulation which lead to irreversible brain injury [9]. Therefore, it is important to enhance acute stroke recovery building upon presently recognized interventions [1,10].

Stem cell therapy (SCT) has been demonstrated to be effective in promoting tissue recovery following ischemic stroke injury, with SCT efficacy depending critically upon the timing and method of administration [2,11]. One of the more widely-applied stem cell types used in SCT are mesenchymal stromal cells (MSCs) [11], [12], [13]. In this study, we tested the therapeutic potential of human placenta-derived mesenchymal stem cells (hPMSCs) in the murine MCAO ischemic stroke model. hPMSCs were chosen because they represent a safe, accessible, abundant, and potentially effective [14,15] form of SCT. It is also viewed as relatively inexpensive and free of ethical concerns.

Historically, stem cells have long been assumed to provide benefit in stroke patients by engrafting within the post-stroke brain where they might trans-differentiate into cells which repair damaged tissue [16]. However, stem cells have also been proposed to protect brain tissue through paracrine signaling which may limit acute brain IRI via barrier-stabilization and suppression of leukocyte adhesion/extravasation mediated tissue injury [12,[17], [18], [19]]. Several lines of evidence now support at least some of these paracrine benefits of SCT as being mediated by extracellular vesicles (EVs) released by stem cells [1,2]. However, currently, the mechanisms by which EVs derived from hPMSCs protect the brain against ischemic insult remain unclear.

We used murine middle cerebral artery occlusion (MCAO) model to monitor changes in infarction size, BBB integrity, and perfusion in the brains of mice with/without hPMSCs and hPMSCs-derived EVs.

We found that intraperitoneal (IP) administration of hPMSCs at the beginning of reperfusion (end of 1-hour ischemia) produced remarkable and highly significant preservation of ipsilateral hemispheric blood flow, tissue structure and neurological recovery following MCAO compared to untreated group. Strikingly, these benefits appear to reflect protective effects of EVs released from hPMSCs. Specifically, these benefits seem to be cholesterol-dependent and related to changes in surface presentation of phosphatidylserine (PS). Based on these lines of evidence, we hypothesize that intraperitoneal (IP) administration of hPMSC provides potent protection against stroke-induced infarction, blood brain barrier failure and neurological deficits by maintaining cerebral perfusion for at least 24 h. We further propose that hPMSC-derived EVs mediate this protection based on: 1) the lack of hPMSC arriving in the bloodstream or brain 2) the ability of cholesterol-lipid supplementation/reduction to influence EV numbers and protection against MCAO and 3) the ability of cholesterol-treated hPMSC to release PS-negative EVs which provide equivalent stroke protection as hPMSC. We conclude that hPMSC and hPMSC/EVs based stroke therapy represents an important procedure that maintains cerebrovascular perfusion and survival.

## 2 Materials and methods

### 2.1 Study design

The objectives of this study were to determine the mechanisms and extent to which hPMSCs protect the brain against acute ischemic injury *in vivo,* and to characterize barrier-stabilizing and anti-inflammatory effects of hPMSCs *in vitro* and *in vivo*. We used the Koizumi method of MCAO as an *in vivo* model of ischemic stroke in C57Bl/6 mice using a 1 h ischemic period following by 24 h reperfusion. hPMSC/EVs were injected (IP/IV) at the time of reperfusion to evaluate how hPMSC/EVs protect against IRI induced by MCAO. A sham group was used as control to evaluate how surgery and anesthesia contribute to observed results. CBF, infarct size, BBB integrity and neurological scores were measured in all experimental groups. We also used oxygen glucose deprivation/reperfusion (OGDR) conditions as our experimental *in vitro* model of ischemic stress where hPMSCs were contact-independently co-cultured with human brain endothelial cell to evaluate protective capabilities of hPMSCs on the *in vitro* barrier generated by human brain endothelial cell monolayers under normoxic and OGDR conditions. In general, we used *n* = 5 to 10 mice per group for in vivo experiments and *n* = 3 for in vitro experiments (with three replicates).

All histopathological analyses and evaluations (Nissl and Iba staining) were accomplished in a blinded fashion. Additionally, immunofluorescent imaging and analyses was performed using an automated evaluation approach (Image-J, NIH). Treatment groups (sham, MCAO groups as well as treatment groups) were performed on same days to help ensure equivalence and reliability. Assignment of hPMSC and/or EVs in these studies was selected based on availability of cells.

### 2.2 Surgery for MCAO model

Male mice (25–30 g) were anesthetized with ketamine (200 mg/kg i.p.)/xylazine (10 mg/kg i.p.). Once under deep anesthesia, middle cerebral artery occlusion (MCAO) was induced by creating a midline incision at the neck to expose the right carotid bifurcation. The right external carotid artery branch was isolated and ligated and the common carotid artery microclipped to permit creation of a small hole in the middle of the common carotid artery. A silicone-coated 6–0-nylon microfilament was introduced into the common carotid artery and the micro-clip released to allow advancement of the filament through the artery until the bulb-tip occluded the origin of the middle cerebral artery (MCA). This filament was left in place for 1 h (ischemia), and reperfusion initiated by withdrawal of the filament. For sham groups, vessels were cleared of overlaying connective tissue (also performed in MCAO) without further manipulation. The wounds were closed using surgical sutures (6–0) and mice allowed to recover from anesthesia. Postoperative monitoring of eating, drinking and movement were performed at 4 and 24 h following recovery.

### 2.3 Neurological testing

Neurological outcomes were evaluated at 4 and 24 h after reperfusion using a 24-point scale (Table S1). Briefly, mice were given positive scores (0–3) for each of the following parameters: 5 min of spontaneous activity, symmetry of movement and forelimbs (outstretching while tail is held), response to vibrissae contact, floor and beam walking, wire cage wall climbing, and reaction to touch on either side of the trunk.

### 2.4 hPMSCs isolation and culture

hPMSCs cells used in this study were isolated as described by [20]. Briefly, Placentas delivered by normal pregnant women were collected immediately after delivery. Since the placenta is considered medical waste, no consent from the patients was required. Villous tissue was separated by sterile dissection from different cotyledons, excluding chorionic and basal plates. After extensive washing with ice-cold phosphate-buffered saline (PBS), villous tissue was digested with trypsin (0.125% trypsin solution containing 0.1 mg/ml DNase I and 5 mM MgCl2) in Dulbecco's Modified Eagle's Medium (DMEM) at 37 °C for 90 min. Digested cells were collected and cultured in DMEM supplemented with 10% fetal bovine serum (FBS). PMSCs started to grow in 3–5 days. At ~80% confluence, the cells were passaged with TrypLE™ Express (Invitrogen, Carlsbad, CA, USA). hPMSCs were characterized using fluorescence-activated cell sorting (FACS) analysis or immunostaining. The primary antibodies used included mouse anti-human CD73 (BD Biosciences; USA), mouse anti-human CD90 (BD Biosciences; USA), mouse anti-human CD34 (BD Biosciences; USA), mouse anti-human HLA-DR (BD Biosciences; USA), mouse anti-human CD44 (Santa Cruz Biotechnology; USA), and mouse anti-human Oct-3/4 (Santa Cruz Biotechnology; USA). CD34-APC served as a negative control. hPMSCs were cultured in Dulbecco's-Modified Eagles's Medium (DMEM; Fisher Scientific; USA) with 10% (w/v) fetal bovine serum (FBS; Gibco; USA) and 1% penicillin/streptomycin (Sigma; USA) and used at passage 3–10. At confluency, hPMSCs cells were washed with PBS/EDTA, detached with 0.25% trypsin (Sigma; USA) for 2 min, and subcultivated at a 1:3 split ratio.

### 2.5 IP injection of hPMSCs

Trypsinized hPMSCs were washed twice with Ca^++^/Mg^++^ free HBSS and centrifuged (1500 RPM, 5 min, 25 °C). 5 × 10^5^ hPMSCs were resuspended in 500μl HBSS solution without Ca^++^/Mg^++^ and injected intraperitoneally (IP) into MCAO-treated mice at reperfusion.

One pre-clinical study suggested a dose of 5 × 10^6^ cells as the maximum number of cells that could be beneficial in rats, with higher doses causing high mortality reflecting emboli [21]. Clinical trials consistently employ 10–20 million cells/kg of body weight [1]. Since this was our first time evaluating protective effects of intraperitoneal injection of hPMSC in our study, we chose a dose near the higher end of the range used in humans and equivalent to that used in the rat study above as our starting reference. Therefore, we injected 5 × 10^5^ cells for 30 g BW (~16.7 million cells/kg).

### 2.6 Inhibition and induction of EVs formation

To investigate effects of cholesterol depletion on hPMSCs-enhanced MCAO outcomes, 10 mM methyl beta-cyclodextrin (MβCD), was added to medium as a non-toxic cholesterol sequestering agent [22,23] for 2 h before harvesting the hPMSCs. Conversely, to enrich hPMSCs cholesterol/lipid content, culture medium was supplemented with synthetic cholesterol (1:250 ratio) (Gibco; USA) and CD lipid concentrate (1:100 ratio) (Gibco; USA) and incubated for 72 h at 37 °C, 5% CO_2_ prior to cell harvesting.

### 2.7 Trypan blue exclusion test of cell viability

To determine the number of viable cells, hPMSC treated with or without MβCD were suspended in PBS containing 0.4% trypan blue in 1:1 ratio and incubated for ~3 min at room temperature. 10 μl of trypan blue/cell mixture was applied to a hemocytometer. The unstained (viable) and stained (nonviable) cells were counted separately in the hemocytometer. To calculate the total number of viable cells per 1 ml of cell suspension, the total number of viable cells was multiplied by 2 (the dilution factor for trypan blue), then multiplied by 10^4^. The percentage of viable cells was calculated as follow: [total number of viable cells per ml/total number of cells per ml (viable+nonviable)]X100.

### 2.8 MTT assay

To evaluate the toxicity of MβCD on hPMSCs, MTT assay was performed [24]. Briefly, hPMSCs were washed with PBS after removal of cell culture media. The cells were incubated at 37 °C with MTT (4,5-dimethylthiazol-2-yl)−2,5-diphenyltetrazolium bromide at the final concentration of 0.5 mg/ml cell culture media for 3 h, when intracellular purple formazan crystals were visible under microscope. MTT was removed and absolute ethanol were added to the cells, followed by 30 min incubation at 37 °C until cells have lysed and purple crystal have dissolved. The absorbance was measured at 570 nm using Synergy H1 Hybrid Reader (BioTek; Vermont, USA). The absorbance reading of the blank was subtracted from all samples, and% viable cells was calculated as follow: [(Abs_MβCD-hPMSC_-Abs_blank_)/(Abs_hPMSC_-Abs_blank_)]X100.

### 2.9 Extracellular vesicle isolation

EVs were isolated as described [25]. Briefly, culture media were collected from confluent hPMSCs 48 h after applying fresh medium. Unattached cells and debris were initially removed by centrifugation at 400x*g* for 10 min (4 °C) and supernatants re-centrifuged at 20,800x*g* for 90 min at 4 °C to pellet EVs. EVs pellets washed twice by centrifugation using 4 °C PBS/1 mM phenylmethylsulfonyl fluoride (PMSF) and pelleted at 20,800 g for 15 min (4 °C). EVs pellets injected intravenously (2 × 10^6^ in 100μl HBSS, Sigma; USA) into mice or evaluated by flow cytometry analysis.

### 2.10 Flow cytometry analysis

To evaluate hPMSC-released EVs by flow cytometry, freshly isolated EVs were resuspended in 100μl Annexin-V Binding Buffer (BD Biosciences, San Jose, CA) and incubated with 5μul of Annexin-V-FITC (BD Biosciences; USA) for 1 h at 4 °C under low-light conditions. 900μl of 1X “Binding Buffer” was added to each sample. These samples were immediately collected on a 4 laser ACEA NovoCyte Quanteon Flow cytometer and data analyzed using NovoExpress 1.2 software. EV flow cytometric analysis was calibrated using Megamix-Plus FSC and Megamix-Plus SSC beads.

### 2.11 Fluorescence activated cell sorting (FACS) of EVs

To study effects of PS negative-EV protection in MCAO, we isolated EVs from cholesterol-treated hPMSCs (described in the ‘Flow cytometry’ section), separated PS negative-EVs by (FACS) based on fluorescent labeling used in MCAO therapy studies.

### 2.12 Cell localization using CytoID tracker

To track hPMSC *in vivo*, hPMSCs were first labelled using CytoID red long-term cell tracer kit (Enzo Life Science; USA). Briefly, cells were trypsinized and labelled with 1 ml of 2x CytoID for 5 min. Staining was stopped by adding 2 ml of stop buffer. Cells were centrifuged (400 g, 5 min), cell pellets resuspended in 10 ml of complete media (DMEM+10% FBS+1% P/S) in a T75 flask and incubated at 37 °C for at least 12 h. CytoID-labelled hPMSCs were prepared and injected as described.

### 2.13 Laser speckle measurement of blood flow

A Perimed Laser Speckle Imager (Pericam PSI HR; Sweden) was used to measure cerebral blood perfusion within the brains of different experimental groups. 24 h after reperfusion, mice under deep anesthesia were placed on a warm pad and the coronal skin removed and perfusion recordings accomplished using a high-resolution Laser Speckle camera (Perimed Laser Speckle Imager) at a working distance of 10 cm. ‘Perfusion’ reflects total cerebral flow signal measured in selected tissue regions of interest. Measurements are expressed as perfusion units (PU), using a fixed scale (arbitrary units).

### 2.14 TTC staining of infarcted tissue

24 h after reperfusion, mice were deeply anesthetized with isoflurane and decapitated. The extent and severity of MCAO was evaluated after removal of the brain and staining of brain slices with 2,3,5-Triphenyltetrazolium chloride (TTC; Sigma; USA) to measure tissue viability and infarct size. After dissection, the brain was immersed in cold PBS for 10 m and sliced into 2.0 mm-thick sections using an anatomical slicer. Brain slices were incubated in 2% TTC/PBS for 30 m at 37 °C. Areas of contralateral, ipsilateral, and infarction in each brain slice were recorded (Nikon 990) and measured using Image-j program (NIH). The infarcted area adjusted to the edema using *Reglodi's method*: (EA)-infarct volume: infarct volume x (contralateral hemisphere/ipsilateral hemisphere) [26]. Cumulative dead (white-stained) regions were combined from each brain to generate a total brain tissue infarcted score for each mouse.

### 2.15 Evans blue vascular permeability evaluation

BBB disruption following MCAO/reperfusion was measured by quantitating Evans blue (EB) transvascular leakage into the brain at 24 h. After MCAO, mice under deep anesthesia were injected with 100μl of 2% EB, (4 mg/kg) through the femoral vein and allowed to circulate for 20 min before sacrifice. 0.2 ml blood was collected from the left ventricle and centrifuged at 5000RPM for 10 min to obtain plasma. Circulating dye was cleared by perfusing mice with 15 ml cold PBS. 10μl plasma (supernatant) was added to 990 μl of 50% trichloroacetic acid (TCA; Sigma; USA), homogenized, sonicated and centrifuged (10,000RPM) for 20 min. To extract EB from brain tissue, 2 ml of 50% TCA solution added to each brain, and the brain/TCA mixture homogenized and sonicated (amplitude 30, 10 W), and centrifuged at 10,000RPM for 20 min and finally diluted 3-fold with 100% ethanol. The amounts of EB in both plasma and brain tissue were quantified at 620 nm excitation and 680 nm emission using Synergy H1 Hybrid Reader (BioTek; Vermont, USA). EB leakage into brain tissue was normalized to the amount of EB in plasma.

### 2.16 Tissue preparation

24 h after reperfusion, mice under deep anesthesia were cleared of blood with 15–20 ml of PBS. Brains were removed and post-fixed overnight in buffered 4% paraformaldehyde at 4 °C. Brains were sectioned (30 um sagittal slices) and mounted on slides.

### 2.17 Immunohistochemistry staining

Following deparaffinization, rehydration and antigen-retrieval with citrate buffer, 30um sagittal slices of brain tissue were incubated with 3% H_2_O_2_ (blocks endogenous peroxidase) and blocked with 1% bovine serum albumin (BSA; Sigma) and 4% normal goat serum in PBS-Triton (0.1%) for 1 h at 25⁰C. The sections were incubated with rabbit anti-Iba-1 antibody (1:1000, Wako Pure Chemical Industries; USA) at 4 °C overnight and treated with 2⁰-biotinylated anti-rabbit IgG (1:200 in 1% BSA/PBST; Vector Laboratories; USA) for 2 h at 25⁰C. The slices were incubated with Avidin Biotin Complex (R.T.U) (LifeSpan BioSciences; USA) reagent for 1 h at 25⁰C followed by peroxidase substrate (Vector Laboratories; USA). Peroxidase activity was visualized with 3-diaminobenzidine. Slides were dehydrated with graded alcohols, cleared with xylene, and cover slipped.

### 2.18 Nissl staining

Tissue was fixed in 4% paraformaldehyde at 25⁰C for 24 h. Sagittal brain sections (30 um) were mounted on slides and Nissl staining performed [27]. Samples were deparaffinized and rehydrated in decreasing ethanol concentrations. Slides were then processed for Nissl staining with thionin for ~5 min at 25⁰C. Slides were dehydrated with graded alcohols, cleared with xylene and coverslipped. Nissl-stained images were recorded at 20X and 40X.

### 2.19 Immunofluorescence (IF) staining of brain tissues

CD31 and Human nuclear marker (Hu-Nu) expression were assessed using fluorescent staining. Paraffinized brain sections were rehydrated and blocked with 1% BSA and 5% goat serum in PBS for 1 h at 25⁰C and incubated with rabbit anti-CD31 (1:100; Abcam), mouse anti-human nuclear antibody (1:100; Millipore) 12 h at 4 °C. Following 4 washes in PBS (10 min), sections were stained with AlexaFluor-488 goat anti-mouse (Life Technologies; USA), AlexaFluor-647 goat anti-rabbit (Life Technologies; USA) for 2 h at 25⁰C. Samples were washed 4X (10 min) and mounted using DAPI/fluoroshield (Sigma; USA). Images were recorded (Nikon Eclipse E600FN, Tokyo, Japan); and processed with ImageJ software.

### 2.20 hCMEC-D3 isolation and culture

The hCMEC/D3 cell line (received from Dr. P.O. Couraud, INSERM) was isolated from temporal lobe microvessels of human tissue which was excised during surgery for control of epilepsy. The primary isolate enriched in cerebral endothelial cells (CECs) were sequentially immortalized by lentiviral vector transduction with the catalytic subunit of human telomerase (hTERT) and SV40 large Tantigen. CEC were then selectively isolated by limited dilution cloning, and clones were extensively characterized for brain endothelial phenotype using endothelial markers including CD34, CD31, CD40, CD105, CD144 and von Willebrand factors [28]. hCMEC-D3 cells were then cultured on collagen-coated plates using endothelial cell medium (EndoGRO; Millipore; USA) supplemented with MV complete culture media kit (Millipore; USA). When hCMEC-D3 cells reached 90% confluency, cells harvested using 0.25% trypsin (Sigma; USA), and centrifuged (1500RPM, 5 min, 25⁰C). Cells were counted and plated at appropriate densities for each experiment.

### 2.21 Oxygen glucose deprivation reperfusion (OGDR)

1 × 10^5 hCMEC-D3 cells were plated and grown to 80% confluency. After changing the media to glucose free DMEM+10% (w/v) FBS+ 1% P/S, the cells were incubated in a hypoxic chamber (1% O2) for 6, 12 or 16 h followed by 24 h reoxygenation in normal complete DMEM+10% FBS+1% P/S (5% O2).

### 2.22 Transwell co-culture model

In this model (Fig. 2B), hCMEC-D3 cells were plated on the bottom chamber of transwell plates (Corning, USA) in 2 ml of complete media and hPMSCs cultured on the upper surface of cell culture inserts with permeable membrane (3 μm pore size). In this contact-independent model, hPMSCs cannot migrate between compartments and do not directly interact with hCMEC-D3 cells.

### 2.23 Biotinylated gelatin/FITC avidin permeability assay

To measure endothelial barrier function following OGDR, we used the biotinylated gelatin/FITC avidin method as described in Ez-link-biotin protocol (Thermo Fischer; USA). Briefly, biotinylated gelatin solution added to 12-well plates and incubated at 4 °C overnight. After removing the biotinylated gelatin solution, hCMEC-D3 were plated at 2 × 10^5^ cells/well. FITC-avidin (1:50), (Life Technologies-Molecular Probes; USA) was added directly to the media and incubated for 3 min at 37 °C under low-light conditions. The cells were washed with 37 °C PBS twice, and fixed with 4% paraformaldehyde for 10 min at 25⁰C. Images were acquired using Nikon video imaging system Eclipse E600FN (Nikon, Tokyo, Japan) at 20X and processed with NIH-ImageJ software.

### 2.24 Western blot analysis

After desired treatments, cells collected in Laemmli buffer (Bio-Rad; USA) containing 10% 2-mercaptoethanol. The cells were scraped and sonicated at power of 50% for 15 sec, boiled at 95 °C for 15 min. 20 μl of protein was separated via SDS-PAGE, then immunoblotted to PVDF and incubated at 4 °C with rabbit anti-ZO-1 (1:500), rabbit anti-α-claudin-1 (1:500), (Invitrogen), rabbit anti-occludin (1:1000), rabbit anti-α-catenin (1:1000,Abcam), rabbit anti-VE-cadherin (1:1000) and rabbit anti-β-tubulin (1:2000,Cell Signaling). Membranes were incubated with goat anti-rabbit IgG-HRP antibodies (1:2500, Sigma) for 2 h at 25 °C. Signal was detected using ChemiDocTM MP imaging system (Bio-Rad) and results analyzed with NIH Image-J software.

### 2.25 IF staining of hCMEC-D3

For IF staining, hCMEC-D3 grown under normoxia/OGDR conditions were co-cultured with and without hPMSCs. The cells were washed with wash buffer (PBS+MgCl_2_+CaCl_2_+protease inhibitor), and then fixed in ice-cold 4% paraformaldehyde for 10 min on ice, and permeabilized (0.5% Triton X-100/PBS, 5 min, 25⁰C). Cells were blocked with 5% BSA/5% goat serum for 1 h at 25⁰C. Primary antibodies (rabbit anti-α-catenin (1:100, Abcam) and rabbit anti-VE-cadherin (1:100, Cell Signaling) were diluted in wash buffer and incubated with cells overnight at 4 °C. Cells were next incubated with fluorescently conjugated secondary antibody (AlexaFluor 488 goat anti-rabbit; Life Technologies; USA) for 1 h and rinsed twice. Hoechst (Thermo Scientific; USA) was added to the cells for 5 min, washed, mounted on glass slides and images recorded using a Nikon video at 20X magnification. Images were processed with Image-J software.

### 2.26 Statistical analysis

Statistical analysis was performed using GraphPad Prism software. For all experiments, data are expressed as mean±standard error of the mean (SEM). The statistical significance of the differences between groups was calculated using Student's *t*-test, one-way ANOVA with Bonferroni post-hoc test or two-way ANOVA with Sidak's multiple comparisons tests where appropriate and indicated in the figure legend. Flow cytometry data were analyzed using Mann Whitney U test. A *p*-value<0.05 was considered statistically significant.

## 3 Ethics statement

**Mice:** All animal protocols were approved by the LSUHSC-S Institutional Animal Care and Use Committee (IACUC) according to NIH guidelines. We used male C57BL/6 J mice (Jackson Laboratories, Bar Harbor, ME) in all studies at 9–16 weeks of age. Animals were housed in a barrier facility and maintained on a normal diet.

**Human Placenta:** Collection of human placentas for MSC isolation was approved by the IRB at Louisiana State University Health Science Center – Shreveport (LSUHSC-S), and MSC isolation was processed at the Department of Gynecology and Obstetrics, LSUHSC-S.

**Human brain endothelial cells (hCMEC/D3):** The hCMEC/D3 cell line was provided by Dr. P.O. Couraud. Cells were isolated from section of brain tissue removed during surgery for epilepsy following informed consent according to protocols established at INSERM, Institut Cochin, France.

National Institute of Health National Institutes of Health grants (NIGMS 5 P20 GM121307–02 Center for Redox Biology and Cardiovascular Disease) provided technical support. The Department of Neurology and Intramural Malcolm Feist Pre-doctoral Fellowship (CCDS-LSUHSC-Shreveport) provided financial support for this study, and did not participate in study design, data collection, data analyses, interpretation, or writing of the paper.

## 4 Data availability

This study did not generate any new unique reagents, datasets or code. Further information and requests for resources and reagents should be directed to the corresponding author, J. Steven Alexander (jalexa@lsuhsc.edu).

## 5 Results

### 5.1 MCAO model of stroke

We induced ipsilateral cerebral hemispheric ischemic injury in C57Bl/6 mice using MCAO model based upon prior studies of this model which were associated with an 88 to 100% rate of infarction [29,30]. Duration of reperfusion is another essential factor influencing pathophysiology and outcomes in the MCAO model [31,32]. TTC staining was used to identify the areas of infarction. Initial studies revealed no consistent infarct pattern 4 h after reperfusion (**Fig. S1a**) which led to the ongoing protocol of one hour of ischemic and then sacrifice of the animal with infarct assessment at 24 h. We observed, in preliminary studies, an infarct volume of 21.94 hours after reperfusion (Fig. S1a) which led to the ongoing protocol of one hour of ischemic and then sacrifice of the animal with infarct assessment at 24h24 hours. We observed, in preliminary studies, an infarct volume of 21.94<span class=``error-correction''>&lt;delete&gt; &lt;delete&gt;</span> \261 0.52%% vs 0 infarct in the sham animals (p<0.0001; Fig. S1b). Reduced CBF following MCAO impairs motor function and survival [null,null]. In agreement with this, we also observed reduced CBF in our MCAO group at 10.69\2611.017 for sham animals compared to 2.723\2610.764 (p=0.0008; Fig. S1c) which was accompanied by substantially decreased neurological scores 5.0\2610.59 in MCAO (impaired motor function) compared to 24.0\2610 in sham mice (p<0.0001; Fig. S1d)."?> ± hours after reperfusion (Fig. S1a) which led to the ongoing protocol of one hour of ischemic and then sacrifice of the animal with infarct assessment at 24h24 hours. We observed, in preliminary studies, an infarct volume of 21.94 \261<span class=``error-correction''>&lt;delete&gt; &lt;delete&gt;</span>\261 0.52%% vs 0 infarct in the sham animals (p<0.0001; Fig. S1b). Reduced CBF following MCAO impairs motor function and survival [null,null]. In agreement with this, we also observed reduced CBF in our MCAO group at 10.69\2611.017 for sham animals compared to 2.723\2610.764 (p=0.0008; Fig. S1c) which was accompanied by substantially decreased neurological scores 5.0\2610.59 in MCAO (impaired motor function) compared to 24.0\2610 in sham mice (p<0.0001; Fig. S1d)."?> 0.52% vs 0 infarct in the sham animals (*p*<0.0001; **Fig. S1b**). Reduced CBF following MCAO impairs motor function and survival [33,34]. In agreement with this, we also observed reduced CBF in our MCAO group at 10.69 ± 1.017 for sham animals compared to 2.72 3 ± 0.764 (*p* = 0.0008; **Fig. S1c**) which was accompanied by substantially decreased neurological scores 5.0 ± 0.59 in MCAO (impaired motor function) compared to 24.0 ± 0 in sham mice (*p*<0.0001; **Fig. S1d**).

### 5.2 Characteristics of hPMSCs

In cell culture environment, hPMSCs are fibroblast-like, “spindle”-shaped, plastic-adherent cells that exhibit robust *in vitro* expansion [35] (**Fig. S2a**). To validate the potential for hPMSCs to replicate stem cell neurorestorative capability, we measured the expression of classical MSC markers CD73, CD90, CD44 and Oct4 by FACS analysis and immunostaining (**Fig. S2b-e**). hPMSCs did not express high levels of HLA-DR, or the hematopoietic lineage marker (CD34) (**Fig. S2f-g**), which is the major criteria to define MSCs.

### 5.3 IP injection of hPMSCs preserves ipsilateral hemisphere viability and perfusion in the MCAO model

**Fig. 1:**
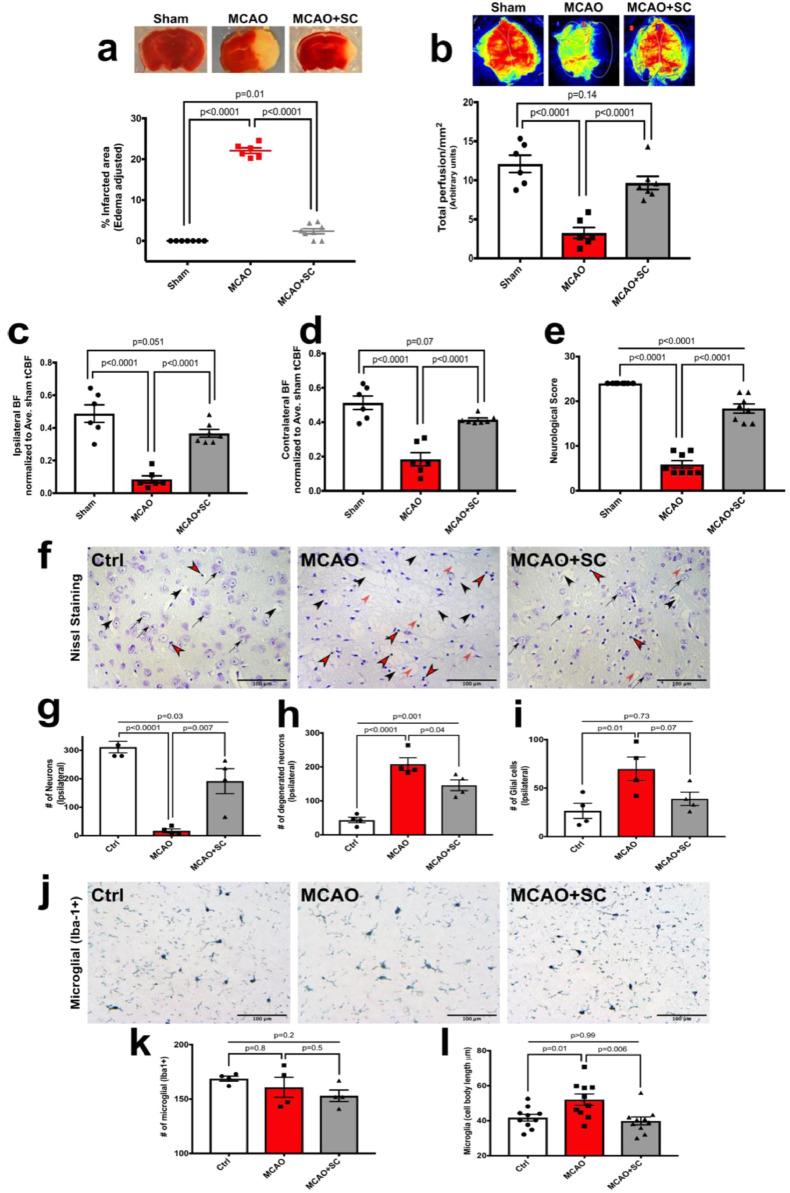
**IP administration of hPMSC protects against ischemic injury in the MCAO stroke model.** (**a**) The area of infarction (‘white’ area) in each brain slice was visualized using TTC staining and measured using Image-J (NIH). Unstained (‘white’) regions in brain slices were combined to generate a composite tissue injury score for each brain. Each point represents one animal (sham (*n* = 7), MCAO (*n* = 6), MCAO+hPMSC (*n* = 8)). Compared to sham, there was a significant increase in infarct size in MCAO mice (^⁎⁎⁎⁎^*p*<0.0001, one-way ANOVA). By comparison infarct size in hPMSC-treated MCAO mice was significantly reduced (^⁎⁎⁎⁎^*p*<0.0001, Student's *t*-test). (**b**) Total cerebral perfusion measured using Laser Speckle imaging. ‘Perfusion’ reflects total cerebral flow signal measured in selected tissue areas. Measurements are expressed as perfusion units (PU), using a fixed scale (arbitrary units). Significant differences were observed between cerebral perfusion in MCAO (*n* = 6) and sham (*n* = 6) mice (^⁎⁎⁎⁎^*p*<0.0001, one-way ANOVA). hPMSC-treated MCAO (*n* = 7) mice showed significant preservation of perfusion compared to non-treated post-MCAO group (^⁎⁎⁎⁎^*P*<0.0001, Student's *t*-test). (**c, d**) Cerebral perfusion in each pair of ipsilateral (**c**) and contralateral (**d**) brain hemispheres were normalized to the average sham total CBF as the reference point. (**c**) A large (83%) decrease in cerebral perfusion of the ipsilateral side of MCAO brain was observed compared to the sham operated group (^⁎⁎⁎⁎^*p*<0.0001, one-way ANOVA); (**d**) A significant decrease (65%) in cerebral perfusion of the contralateral hemisphere of MCAO brain was observed compared to the sham group (^⁎⁎⁎⁎^*p*<0.0001, one-way ANOVA). hPMSC treatment significantly preserved blood flow normal distribution between the hemispheres (far right bars; **c, d**); significant differences determined by Student's *t*-test, ^⁎⁎⁎⁎^*p*<0.0001 and ^⁎⁎⁎⁎^*p*<0.0001 for comparison of hPMSC-treated MCAO mice to untreated MCAO ipsilateral (**c**) / contralateral (**d**) hemispheres, respectively. (**e**) Neurological scores 24 h post-reperfusion. Significant differences in neurological scores were seen between untreated MCAO (*n* = 8) and hPMSC-treated MCAO (*n* = 8) to sham (*n* = 8) group (^⁎⁎⁎⁎^*p*<0.0001, one-way ANOVA). There was a significant improvement in neurological score in hPMSC-treated MCAO mice compared to untreated MCAO (^⁎⁎⁎⁎^*p*<0.0001, Student's *t*-test). (**f**) Neuronal degeneration in MCAO with hPMSC therapy. Neurons (black arrows), degenerating neurons (black arrows head), glial cells (red arrows head), and spongiform regions (red arrows) were visualized using Nissl staining of brain sections in untreated and hPMSC-treated MCAO groups. Two-tailed Student's *t*-test analysis revealed significant differences in proportions of (**g**) neurons (*p = 0.02), (**h**) degenerating neurons (*p = 0.04), and (**i**) glial cell (*p = 0.02) numbers in hPMSC-treated MCAO brain sections compared to untreated MCAO sections. In all graphs, data represent means ± SEM. (**j**) Representative images after immunohistochemistry staining for Iba-1. (**h**) Quantification of numbers of Iba-1^+^ microglia in the border between striatum and cortex (average of 3 different fields) of ipsilateral hemisphere of MCAO and MCAO+hPMSC groups (NS, *p* = 0.5, Student's *t*-test, *n* = 4 per group). (**i**) Microglial activation in ipsilateral hemispheres of MCAO and MCAO+hPMSC groups was determined by cell body length (^⁎⁎^*p* = 0.002, Student's *t*-test). (For interpretation of the references to color in this figure legend, the reader is referred to the web version of this article.)

We assessed the potential of IP injection of hPMSCs, upon cerebral reperfusion, helps to preserve cerebral tissue integrity following the ischemic insult. Strikingly, using TTC staining, we observed that IP administration of 5 × 10^5^ hPMSCs significantly reduced ischemic injury in our model (2.37 ± 0.74%; *p*<0.0001) towards levels observed in sham (0 ± 0) compared to MCAO without SCT (22.08 ± 0.79%) (Fig. 1**a**).

To determine the extent of hPMSCs ability to preserve CBF at 24 h after IP-SCT in the MCAO mice, we measured cerebral perfusion using Laser Speckle Contrast Imaging [36]. There was significant preservation of CBF in our hPMSC-treated group (9.657 ± 0.85 vs 3.24 ± 0.72 in untreated post-MCAO) (*p* = 0.0001; Fig. 1**b**). Normalizing either ipsilateral or contralateral blood flow to the average sham total CBF value as the reference point, revealed significant decreases in both ipsilateral (0.08 ± 0.02; *p*<0.0001; Fig. 1**c**) and contralateral (0.18 ± 0.04; *p*<0.0001; Fig. 1**d**) hemispheres of untreated MCAO animals. Most interestingly, when comparing CBF between both hemispheres, there was a significant shift in the balance of blood flow towards the contralateral hemisphere of MCAO mice. As shown in **Fig. S3,** blood perfusion in both hemispheres of the sham group was comparable (51.71% in contralateral vs. 49.17% in ipsilateral; *p*>0.99). This balance significantly shifted towards the contralateral hemisphere in the MCAO group (20% in contralateral vs. 6% in ipsilateral; *p* = 0.04). Remarkably, MCAO-induced blood flow disturbances were restored with hPMSC treatment (35% in contralateral vs. 29% in ipsilateral; *p* = 0.5), indicating a redistribution of CBF associated with improved post-MCAO tissue survival in hPMSC-treated mice.

Consistent with abundant evidence showing that reduced CBF after MCAO chronically impairs neural function and survival [37], [38], [39], [40], we also observed that neurological function was significantly maintained in IP-hPMSC treated MCAO mice (18.38 ± 1.01) compared to untreated MCAO group (5.87 ± 0.83) (*p*<0.0001; Fig. 1**e**).

Destruction of neurons is another hallmark of ischemic stroke injury which may be improved by SCT [41,42]. To discriminate viable from degenerating neurons and glia in the striatum of both ipsilateral and contralateral hemispheres, we performed modified Nissl staining (Fig. 1**f-i**). Nissl staining revealed reduced numbers of viable neurons in the ipsilateral hemisphere compared to the contralateral side (16.75 ± 7.12; *p* = 0.02) of MCAO group. This degree of neuronal injury was attenuated by IP-hPMSC following MCAO (191.5 ± 43.91) (black arrows; Fig. 1**f**, quantification; Fig. 1**g**). In comparing ischemic damaged neurons with enlarged intercellular spaces, reflected of cellular destruction in stroke, there was significant reduction in degree of insult with hPMSC-treated mice (146 ± 15.83 vs 208.3 ± 18.81; *p* = 0.04) (black arrows head; Fig. 1**f**, quantification; Fig. 1**h**). Many unstained (‘spongiform’) regions were also observed in MCAO-treated brains; this appearance was not detected in any IP-hPMSC treated MCAO brains (red arrows; Fig. 1**f**). In addition, the proliferation of reactive glial cells (red arrows head; Fig. 1**f)**, was reduced from 69.75 ± 12.36 in the untreated ipsilateral hemispheres of MCAO mice compared to 39 ± 6.86 with IP-hPMSC (*p* = 0.02, quantification; Fig. 1**i**).

Early activation of microglia (resident immune cells in the CNS) [43,44], is a key neuroimmunological responses to a wide variety of pathological stimuli e.g., trauma, inflammation, degeneration, ischemia [45,46]. Ionized calcium binding adaptor protein (Iba-1), is specifically mobilized in microglia after inflammation and plays important roles in microglial regulation/activation [45,47,48]. In recognition that inflammatory cell invasion is part of the pathogenesis of evolving cerebral ischemia, we assessed microglia activation via immunohistological staining with anti-Iba-1 antibody (Fig. 1**j-l**). Iba-1^+^ microglia were counted in the border areas between cortex and striatum of each hemisphere. We found the number of microglia (Iba-1^+^) within the ipsilateral hemispheres with administration of hPMSCs following MCAO, was similar to that in the MCAO group, 153 ± 5.33 and 160.8 ± 9.31, respectively (*p* = 0.5) (Fig. 1**j, k**). However, elongated microglial cell bodies observed in MCAO mice (52.02 ± 3.23) was significantly (*p* = 0.002) reduced by IP-SCT (39.84 ± 2.23) (Fig. 1**j, l**). This supports hPMSCs administration inhibiting microglial activation with presumably a salutary effect on the ischemic insult.

### 5.4 hPMSCs maintain blood brain barrier integrity against MCAO in mice

**Fig. 2:**
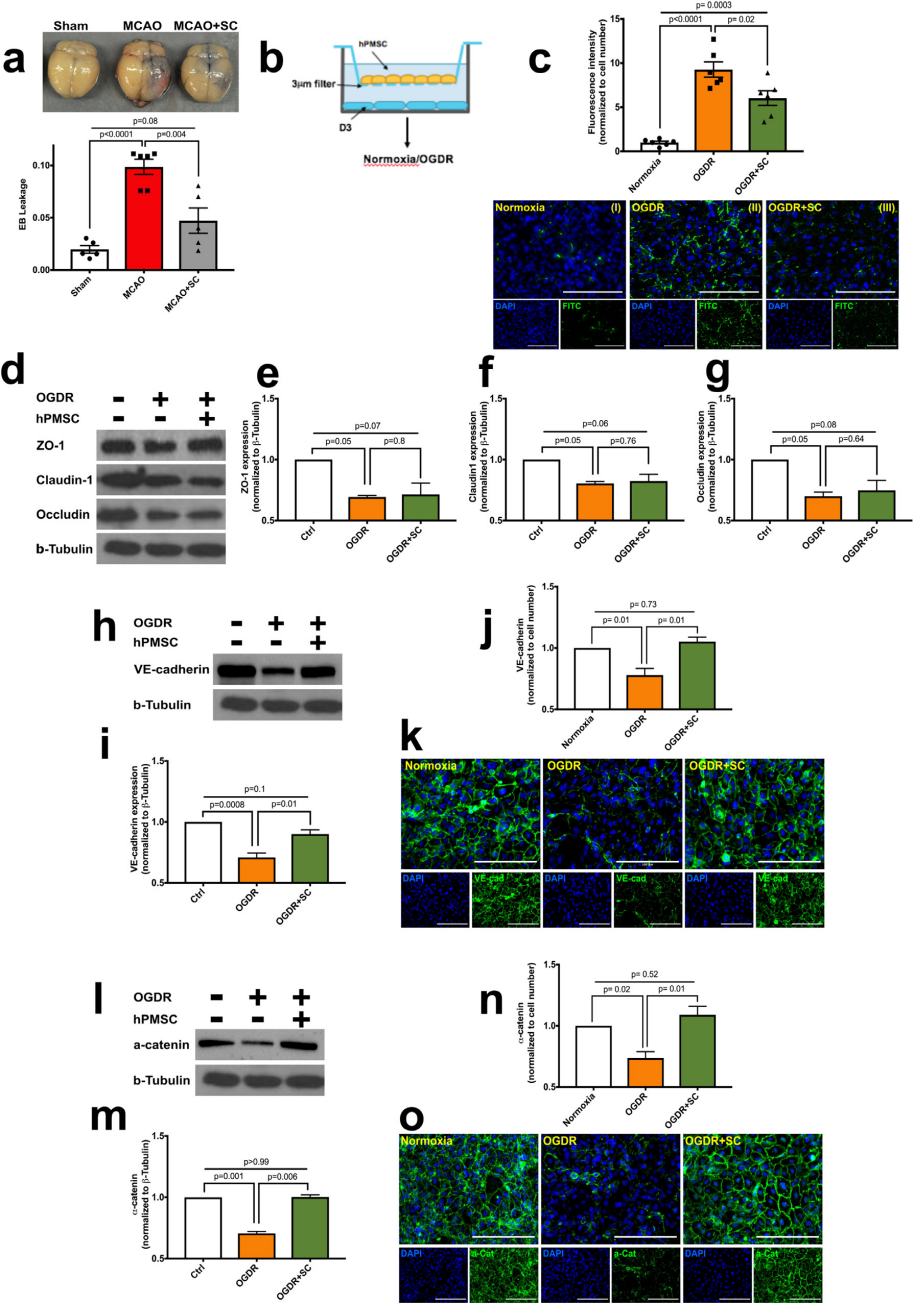
**hPMSCs maintain blood brain barrier integrity against MCAO in mice.** (**a**) BBB disruption assessed by Evans blue (EB) leakage into the brains of sham (*n* = 4), MCAO (*n* = 6), hPMSC-treated MCAO (*n* = 5) mice 24 h following reperfusion. Significant differences in EB leakage were observed between MCAO versus sham (^⁎⁎⁎⁎^*p* = 0.0001, one-way ANOVA) and hPMSC-treated MCAO mice (^⁎⁎⁎^*p* = 0.004, Student's *t*-test). No significant differences were detected between hPMSC-treated MCAO versus sham (NS, *p* = 0.08, one-way ANOVA). (b) Schematic of in vitro model of ischemic stress. (**c**) Barrier function of human brain endothelial (hCMEC-D3) monolayers under normoxia and oxygen-glucose deprivation reperfusion (OGDR) condition were measured using biotinylated-gelatin/FITC-avidin evaluation. Significant differences in fluorescence intensity of D3 monolayers were observed under normoxia versus OGDR (^⁎⁎⁎⁎^*p*<0.0001, One-way ANOVA) and OGDR versus OGDR+hPMSC (*p = 0.02, Student's *t*-test). (**I to III**) Representative images of biotinylated-gelatin FITC-avidin permeability assay of monolayers under (I) normoxic, (II) OGDR, and (III) OGDR+hPMSC conditions. Scale bars, 100 μm (I to III). (**d**) Western blots for expression of ZO-1, Claudin-1, Occludin, and β-tubulin in D3 monolayers under normoxia and OGDR (with/without hPMSC). Quantification of ZO-1 (**e**), claudin1 (**f**), and occludin (**g**) expression normalized to β-tubulin protein expression. No significant differences were observed in the expression of ZO-1 (**e**; *p* = 0.8), claudin1 (**f**; *p* = 0.76) or occludin (**g**; *p* = 0.64) in monolayers under OGDR condition versus OGDR+hPMSC group using two-tailed Student's *t*-test analysis. Immunoblot and quantification of VE-cadherin (**h, i**) and α-catenin (**l, m**) protein expression normalized to β-tubulin. Significant differences were found between OGDR versus OGDR+hPMSC (**i;**^⁎⁎^*p* = 0.01 and **m;**^⁎⁎⁎^*p* = 0.006, Student's *t*-test). Immunofluorescence staining and quantification of VE-cadherin (**j, k**) and α-catenin (**n, o**). Fluorescence intensity of VE-cadherin and α-catenin (green color; **k** and **o**, respectively) was normalized to cell numbers (DAPI-stained nuclei, blue color). Significant differences in fluorescent intensity for VE-cadherin (**j**; ^⁎⁎^*p* = 0.01, Student's *t*-test) and α-catenin (**n**; ^⁎⁎^*p* = 0.01, Student's *t*-test) were observed in hPMSC-treated monolayers under OGDR. Scale bars, 100 μm (**k** and **o**). (For interpretation of the references to color in this figure legend, the reader is referred to the web version of this article.)

To characterize changes in BBB integrity in MCAO-stressed brains, Evans blue (EB) vascular permeability analysis was performed. Fig. 2**a** shows that EB leakage significantly increased in the ipsilateral hemisphere of MCAO group (0.09 ± 0.007 vs sham:0.02 ± 0.01; *p* = 0.0001) shown by blue tissue stain (Fig. 2**a**); indicating that BBB function was lost. In contrast, 24 h after IP administration of 5 × 10^5^ hPMSCs, BBB integrity was maintained as shown by low EB uptake (0.04 ± 0.01; *p*<0.0001) into the ipsilateral hemisphere compared to MCAO group (Fig. 2**a**).

We previously showed that oxygen glucose deprivation (OGDR) increases endothelial permeability [49,50]; similar stresses in stroke may impair BBB function. We therefore investigated whether hPMSCs could maintain the *in vitro* barrier formed by human brain endothelial cell against OGDR (**schematic** Fig. 2**b**). Using a biotinylated gelatin FITC-avidin permeability assay we found that OGDR significantly increased FITC-avidin permeability (9.26 ± 0.87, *p* = 0.0001; Fig. 2**c**), and that hPMSCs contact-independently stabilized hCMEC-D3 barrier integrity against OGDR stresses (6.03 ± 0.83, *p* = 0.02; Fig. 2**c**).

We reported that increased endothelial permeability following ischemia reflects alterations in organization of tight/adherens junctional (TJs/AJs) proteins (e.g. occludin, claudins, VE-cadherin, catenins) [50]. We assessed the impact of OGDR on TJ/AJ protein expression at 6, 12 and 16 h. **Fig. S4a-f** reveals significant reductions in the abundance of ZO-1; (*p* = 0.03), Claudin-1; (*p* = 0.02), Occludin; (*p* = 0.0005; **Fig. S4a-c**) and VE-cadherin; (*p* = 0.05), α-catenin; (*p* = 0.02; **Fig. S4d-e**) after 16 h incubation of hCMEC-D3 monolayers under OGDR stress. We chose 16 h time point of OGDR for following *in vitro* experiments. We next evaluated whether hPMSCs could prevent loss of TJs/AJs in hCMEC-D3 monolayers under both normoxia and OGDR. The expression level of tight junctional proteins e.g., ZO-1 (*p* = 0.8; Fig. 2**d, e**), claudin-1 (*p* = 0.76; Fig. 2**d, f**) and occludin (*p* = 0.64; Fig. 2**d, g**) did not change significantly under OGDR (Fig. 2**d-g**) nor normoxia (**Fig. S5a-c, f**) with hPMSCs. However, we found that hCMEC-D3 monolayers expressed significantly more VE-cadherin (*p* = 0.01; Fig. 2**h, i**) and α-catenin (*p* = 0.006; Fig. 2**l, m**) under OGDR conditions when contact-independently co-cultured with hPMSCs); this was not observed with stem cells under normoxic conditions (**Fig. S5d-f**). The spatial localization of VE-cadherin (**k**) and α-catenin (**o**) in D3 monolayers improved with hPMSC treatment under OGDR stress. Additionally, VE-cadherin (*p* = 0.01; Fig. 2**j, k**) and α-catenin (*p* = 0.01; Fig. 2**n, o**) abundance increased in hCMEC-D3 junctions under OGDR at 24 h after hPMSCs treatment. Taken together, our findings indicate that hPMSC factors improve brain endothelial barrier function by potentially enhancing endothelial junctions after ischemic stress.

### 5.5 hPMSC-released EVs contribute to SC-based protection in the MCAO model

To determine numbers of hPMSCs in the bloodstream, CytoID-dye labeled hPMSCs (**Fig. S6a**) were IP-injected into the MCAO mice, and blood collected at 2, 6, and 24 h post-injection. Interestingly, we found extremely low numbers of circulating hPMSCs (maximum of 2300 cells in 1 ml blood at 6 h; **Fig. S6b**). We next examined brain tissue for the presence of hPMSCs using anti-human nuclear antibody to detect hPMSCs; mouse CD31 was used as a positive control. We did not detect any hPMSCs penetrating the brain (**Fig. S6c**), consistent with beneficial effects of hPMSCs mediated by paracrine signaling pathways restricted to the vasculature rather than cell-integration into the brain.

**Fig. 3:**
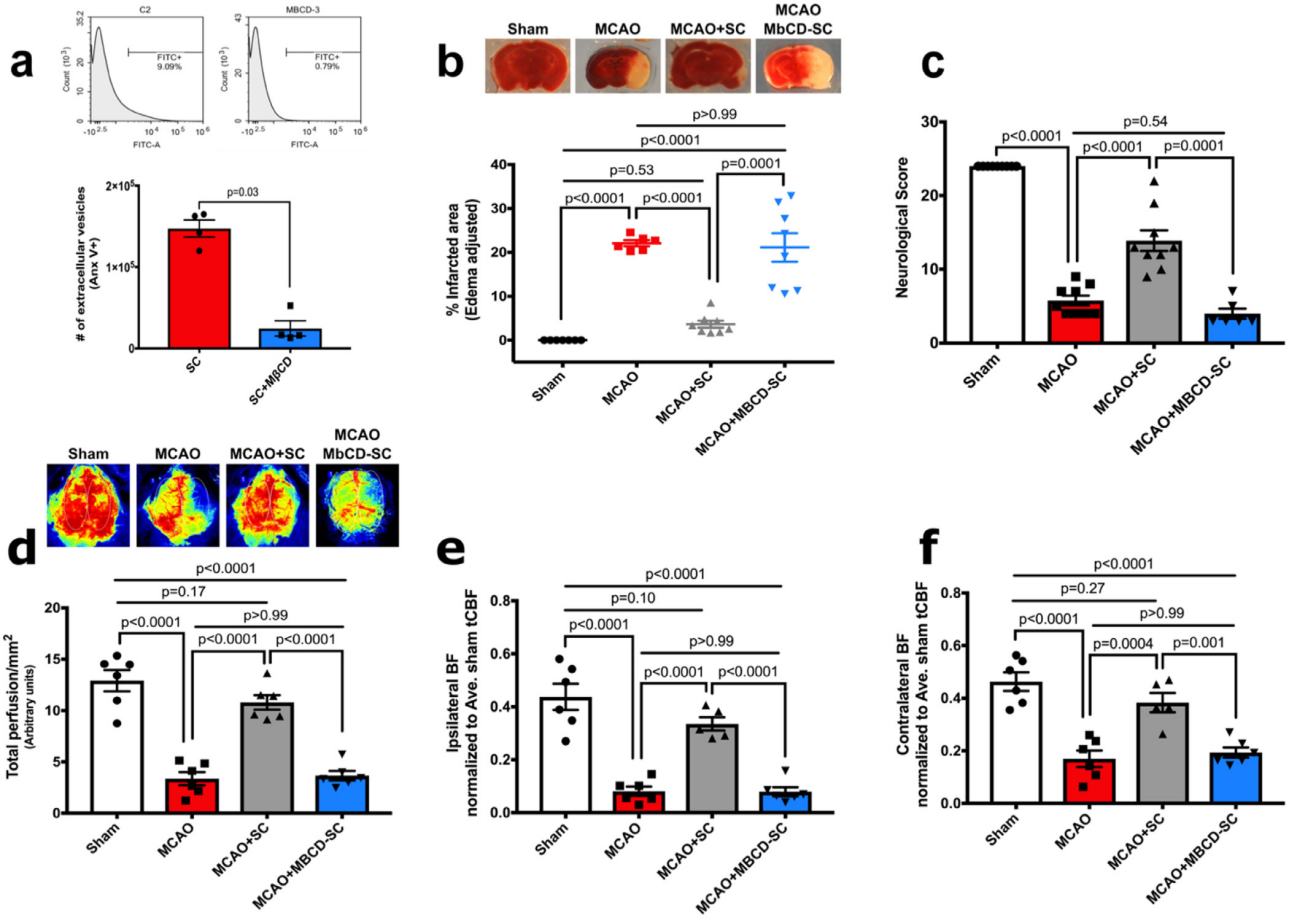
**hPMSC-released EVs contribute to SC-based protection in MCAO.** (**a**) Flow cytometry was used to evaluate numbers of extracellular vesicles (EVs) released from hPMSC treated with or without (10 mM) methyl-beta cyclodextrin (MβCD) for 2 h. Mann Whitney *U test* analysis revealed significant differences in numbers of EVs released from hPMSC compared to MβCD-treated hPMSC (^⁎⁎^*p* = 0.03). (**b**) Infarcted areas were assessed by TTC staining. Significant differences of infarction were measured between MCAO (*n* = 6) mice versus MCAO+hPMSC (*n* = 8) (^⁎⁎⁎⁎^*p*<0.0001) and MCAO+*M*βCD-treated hPMSC (*n* = 8) (NS, *p*>0.99) using one-way ANOVA. Significant increases in the infarcted area were observed in MCAO+*M*βCD-treated hPMSC vs. MCAO+hPMSC mice (^⁎⁎⁎⁎^*p* = 0.0001, Student's *t*-test). (**c**) Neurological scores of MβCD-treated hPMSC (*n* = 6) mice were comparable to MCAO (*n* = 9) group (NS, *p* = 0.54, one-way ANOVA). Significant differences of neurological scores were observed in MCAO+hPMSC mice versus MCAO group (^⁎⁎⁎⁎^*p*<0.0001; one-way ANOVA) and MβCD-treated hPMSC mice (^⁎⁎⁎⁎^*p* = 0.0001; Student's *t*-test). (**d**) Significant differences in brain perfusion of MCAO+hPMSC (*n* = 6) mice subjected to MCAO (*n* = 6) (^⁎⁎⁎⁎^*p*<0.0001, one-way ANOVA) and those given MβCD-treated hPMSC (*n* = 6) (^⁎⁎⁎⁎^*p*<0.0001, Student's *t*-test) group were measured. No significant differences were detected in MCAO group compared to MβCD-treated hPMSC group (NS, *p*>0.99, one-way ANOVA). (**e**) Significant decreases in blood flow into the ipsilateral hemisphere of MCAO (^⁎⁎⁎⁎^*p*<0.0001, one-way ANOVA) and MCAO+*M*βCD-treated hPMSC (^⁎⁎⁎⁎^*p*<0.0001, Student's *t*-test) treated mice were compared to hPMSC-treated MCAO groups. Changes in the ipsilateral perfusion of MCAO and MCAO+*M*βCD-treated hPMSC groups were not significant (NS, *p*>0.99, one-way ANOVA). (**f**) Significant decreases in blood flow into the contralateral hemisphere of MCAO (^⁎⁎⁎⁎^*p* = 0.0004, one-way ANOVA) and MCAO+*M*βCD-treated hPMSC (^⁎⁎⁎^*p* = 0.001, Student's *t*-test) brains was detected compared to MCAO+hPMSC groups. No significant differences of contralateral perfusion were observed between MCAO and MCAO+*M*βCD-treated hPMSC mice (NS, *p*>0.99, one-way ANOVA). All graph data show the means ± SEM.

Several recent studies have suggested that extracellular vesicles (EVs) derived from stem cells contribute at least in part to the ‘paracrine’ arm of SC-mediated benefits in stroke [51], [52], [53]. EVs are biological vesicles released by cells that contain molecules involved in cell communication, repair and differentiation [51,54]. Cholesterol is an important structural component of EVs and also regulates EVs functional properties [55]. To investigate the possible participation of hPMSC-derived EVs in the enhanced MCAO outcomes seen with hPMSC administration, we blocked the formation/release of EVs from stem cells using 10 mM methyl-beta cyclodextrin (MβCD), which is known to be a non-toxic cholesterol chelating agent [22,23]. We did not observe morphological changes in MβCD-treated hPMSCs (**Fig. S7a**); trypan blue staining found no differences in cell viability (*p* = 0.28**; Fig. S7b**). Further, to detect cell stress in response to exposure to MβCD, we also performed MTT staining on MβCD-treated hPMSCs. We observed that MβCD-treated hPMSC metabolized MTT similar to control hPMSCs (*p* = 0.98**; Fig. S7c**), consistent with 10 mM MβCD not provoking toxicity within hPMSCs after 2 h incubation time. Lastly, flow cytometric analysis showed a significant decrease (*p* = 0.03, Mann Whitney U test) in the numbers of EVs released from MβCD-treated hPMSCs compared to untreated hPMSCs (Fig. 3**a**). Unlike hPMSC treatment, we observed that MCAO mice injected with MβCD-treated hPMSCs (21.11 ± 2.63) failed to show protection against MCAO injury (22.08 ± 2.83, *p*>0.99; Fig. 3**b**). Similarly, neurological deficits (4 ± 1.77, *p* = 0.54; Fig. 3**c**) and CBF reductions (3.66 ± 1.05, *p*>0.99; Fig. 3**d-f**) were similar to untreated MCAO (neurological score: 5.77 ± 1.19 and blood flow: 3.36 ± 1.05). Therefore, hPMSC-derived EVs appear to represent critical mediators of SC-based protection after stroke.

### 5.6 Cholesterol/lipid supplementation augmented protective potential of hPMSCs in the MCAO mice

**Fig. 4:**
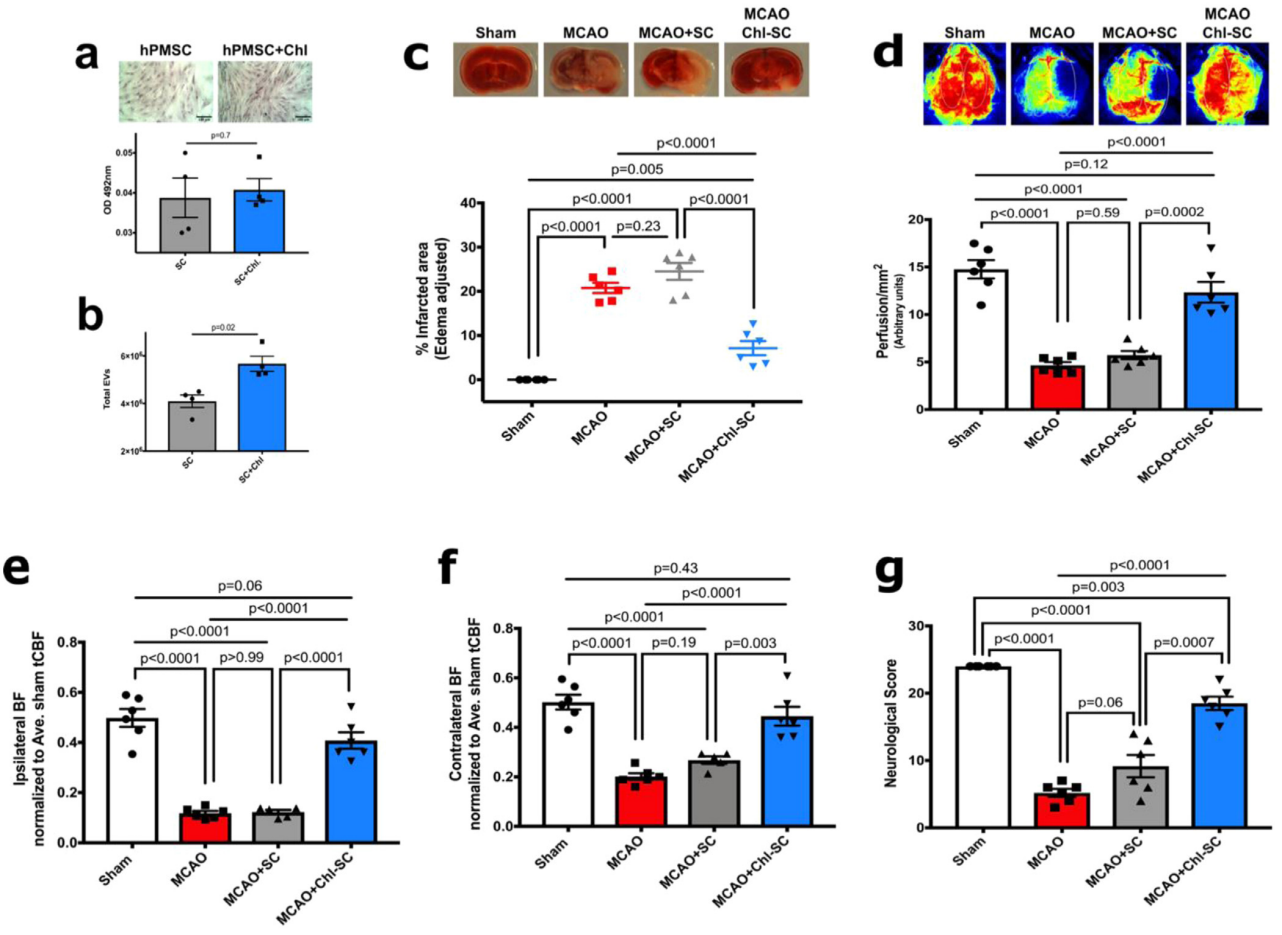
**Cholesterol/lipid supplementation enhances the protective capacity of hPMSCs in MCAO.** (**a**) Cholesterol-lipid content of hPMSCs was evaluated using Oil Red O staining which stains lipid droplets and intracellular cholesterol. No significant changes in intracellular cholesterol/lipid were observed (NS, *p* = 0.7, Student's *t*-test). (**b**) Numbers of EVs released from hPMSCs and cholesterol-treated hPMSCs were calculated using flow cytometric analysis. Significant increases in numbers of EVs released from cholesterol-treated hPMSCs were found compared to untreated hPMSCs (^⁎⁎^*p* = 0.02, Mann Whitney *U* test). (**c-g**) Protective potential of 1 × 10^5^ hPMSCs and cholesterol-treated hPMSCs were compared in MCAO. Significant differences of (**C**) infarcted area (TTC staining; NS, *p* = 0.23 and ^⁎⁎⁎⁎^*p*<0.0001), (**d**) total cerebral blood flow (NS, *p* = 0.59 and ^⁎⁎⁎⁎^*p* = 0.0002), and (**g**) neurological scores (NS, *p* = 0.06 and ^⁎⁎⁎⁎^*p*<0.0001) were measured using one-way ANOVA to compare MCAO (*n* = 5) with MCAO+hPMSC (*n* = 6) and MCAO+Chl-hPMSC (*n* = 6) groups*.* (**e**) Ipsilateral perfusion improved in MCAO+Chl-hPMSC mice compared to MCAO (^⁎⁎⁎⁎^*p*<0.0001, one-way ANOVA) and MCAO+hPMSC (^⁎⁎⁎⁎^*p*<0.0001, Student's *t*-test) groups. Significant decreases in perfusion into ipsilateral hemispheres of both MCAO (^⁎⁎⁎⁎^*p*<0.0001) and MCAO+hPMSC (^⁎⁎⁎⁎^*p*<0.0001) groups versus sham mice was observed (one-way ANOVA). (**f**) Significantly decreased blood flow in contralateral hemispheres in MCAO (^⁎⁎⁎⁎^*p*<0.0001) and MCAO+hPMSC (^⁎⁎⁎⁎^*p*<0.0001) was observed (versus sham). Redistribution of contralateral perfusion in MCAO+Chl-hPMSC mice was comparable to sham (NS, *p* = 0.43, one-way ANOVA) groups. No significant differences were detected in ipsilateral (**e**; *p*>0.99) or contralateral (**f**; *p* = 0.19) hemispheres of MCAO mice compared to MCAO+hPMSC mice (by one-way ANOVA.) All graph data show means ± SEM. (For interpretation of the references to color in this figure legend, the reader is referred to the web version of this article.)

To test how cholesterol status might contribute to the formation and release of EVs, we treated hPMSC with cholesterol-lipid supplemented media and evaluated the cholesterol content of these cells using Oil Red-O staining. We did not find changes in the lipid and/or intracellular cholesterol content in treated cells (*p* = 0.7; Fig. 4**a**), however, flow cytometry did reveal significant increases in the numbers of EVs released from hPMSCs by 72 h after cholesterol-lipid treatment (*p* = 0.02; Fig. 4**b**). This suggests that cholesterol provided to these cells may be processed into EVs, which was observed by flow cytometry analysis. To evaluate roles of cholesterol-lipid supplementation in hPMSC-mediated protection, 1 × 10^5^ cholesterol-supplemented hPMSCs or non-supplemented cells were IP-injected into MCAO treated mice upon reperfusion after 1-hour ischemia. Strikingly, this lower number of cholesterol-supplemented hPMSCs (20% of the number used previously) (Fig. 1) still significantly prevented MCAO brain injury (7.13 ± 2.24, *p* < 0.0001), while the same number of untreated hPMSC (1 × 10^5^) was insufficient to provide protection (24.52 ± 2.24, *p* = 0.23; Fig. 4**b**). Behavioral performance (18 ± 1.65, *p*<0.0001; Fig. 4**c**) and blood perfusion (12.34 ± 0.99, *p*<0.0001; Fig. 4**d-f**) were also significantly improved in the low (20%) dosed cholesterol-lipid supplemented hPMSC group. Consequently, cholesterol-lipid supplementation was found to significantly enhance the protective capacity of hPMSC in MCAO, apparently through greater formation/release of EVs.

**Fig. 5:**
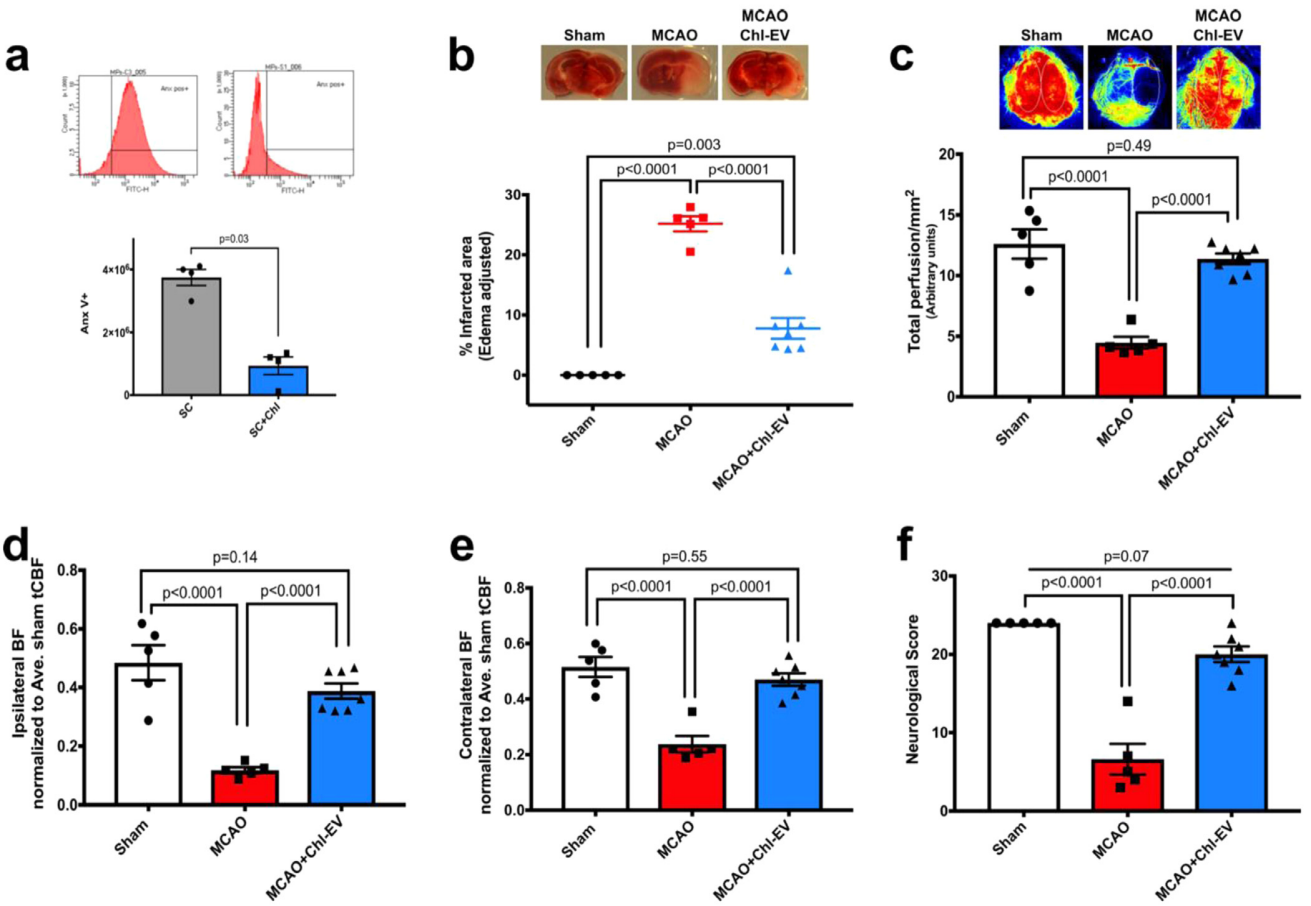
**IV injection of EVs derived from Chl-treated hPMSCs is protective in MCAO.** (**a**) Flow cytometric analysis showed that Chl-treatment significantly reduced phosphatidylserine (PS) expression (decreased Annexin-*V*^+^) on EVs (^⁎⁎^*p* = 0.03, Mann Whitney *U* test). (**b-e**) Protective potency of IV injection of Chl-treated hPMSC-EVs (2 × 10^6^ EVs in 100 μl HBSS) was evaluated in MCAO. Significant differences in infarcted area (**b**; ^⁎⁎⁎⁎^*p*<0.0001), total cerebral perfusion (**c**; ^⁎⁎⁎⁎^*p*<0.0001), ipsilateral perfusion (**d**; ^⁎⁎⁎⁎^*p*<0.0001), contralateral perfusion (**e**; ^⁎⁎⁎⁎^*p*<0.0001) and neurological scores (**f**; ^⁎⁎⁎⁎^*p*<0.0001) of MCAO+Chl-treated EVs (*n* = 7) and untreated MCAO (*n* = 5) groups were identified using Student's *t*-test analysis. The above parameters: **c**; total perfusion (*p* = 0.49), **d**; ipsilateral perfusion (*p* = 0.14), **e**; contralateral perfusion (*p* = 0.55), **f**; neurological scores (*p* = 0.07)) in MCAO+Chl-treated EVs mice were comparable to the sham group (one-way ANOVA). All graphed data show means ± SEM.

Interestingly, flow cytometric analysis of EVs collected from cholesterol-hPMSCs revealed a significant decrease in annexin-V *positive* EVs (*p* = 0.03; Fig. 5**a**), indicating a net reduction in phosphatidylserine (PS) presentation on EVs outer surface. Prior studies confirm PS as a potent pro-coagulant factor [56], [57], [58], [59], which recruits coagulation activators e.g. tissue factor (TF) that activate factor X to generate thrombin [60,61]. Such induction of coagulation may explain in part our low rate of survival (15%) after IV injection of untreated hPMSC-derived EVs into MCAO mice (*n* = 7) (**Table S2**). To study whether cholesterol supplementation of hPMSCs might overcome IV-related complications, PS^−^EVs, derived from cholesterol-treated hPMSCs (2 × 10^6^) (i.e. EVs that did not bind Annexin-V) were sorted using FACS and IV-injected into MCAO mice. We next investigated the effect of cholesterol supplementation on the safety and benefits of hPMSCs derived EVs by comparing the MCAO group with MCAO mice IV-injected with EVs isolated from cholesterol-treated hPMSCs (PS^−^EVs). By comparison 80% of mice (*n* = 10) IV injected with PS^−^EVs survived (**Table S2**). Fig. 5 shows that tissue injury was significantly reduced from 25.17 ± 1.25 in MCAO to 7.76 ± 1.73 in the cholesterol-treated hPMSC PS^-^ EV group with MCAO (*p*<0.0001; Fig. 5**b**); blood perfusion (11.38 ± 0.44, *p*<0.0001; Fig. 5**c-e**) and behavior (20 ± 1, *p*<0.0001; Fig. 5**f**) were both significantly improved.

## 6 Discussion

Stroke remains the leading cause of neurologically-based morbidity worldwide [2] with thromboembolic strokes accounting for 87% of total stroke incidence. In ischemic stroke, IRI at the time of, and following therapeutic restoration of CBF often mobilizes intracerebral inflammatory mediators that impair BBB, activate endothelial cells and disturb normal CBF, all of which greatly intensify stroke severity [49]. Pharmaceutical interventions for stroke are now limited to two FDA-approved approaches: t-PA and anti-platelet therapies (aspirin/clopidogrel). While these treatments aim to restore blood flow to the brain, their clinical use does not halt the initiation and progression of cerebral reperfusion injury and each carries serious risks for hemorrhage [62]. The lack of highly effective and safe therapies for the acute phase of stroke still demands investigation towards alternative therapeutic approaches for limiting stroke injury using stroke models e.g. MCAO [10,63].

Presently, MSCs from many adult and fetal tissues [11,12] and blood, are being intensively studied as powerful and promising tools in ischemic stroke therapy both in clinical and preclinical trials; their properties and therapeutic applications vary widely depending on their source, isolation procedures, time and route of administration [13,63,64]. The placenta represents an important and highly practical source of MSC for SCT. Placentas contain extremely high numbers of stem cells, with no need for invasive recovery procedures; placentas also lack ethical considerations encountered with fetal tissues [14,15].

Here, our hPMSC were plastic adherent, CD34^(-)^, CD10^(+)^, CD200^(+)^ and CD105^(+)^ cells (**Fig. S2**). In setting up our study, we considered potential difficulties associated with xenografting, where host immune response could reject cells from non-human sources (such as murine or porcine cells) [65]. Several studies have shown that human stem cells *can* improve ischemic outcomes in preclinical *animal* models [36,66,67]. In fact, reports of the immunomodulatory effects and low immunogenicity of human placental derived stem cells (hPMSC) found these cells to be useful candidates for allogenic transplantation and clinical application in regenerative medicine [68]. Therefore, if these cells were effective in animal models, it is likely they could have benefits in human clinical treatment.

While SCT is commonly used chronically, early and effective limitation of initial stroke injury still represents the greatest opportunity to successfully manage injury, thereby limiting the degree of tissue repair that would otherwise be required later. However, less is known about the benefits of *acute* (vs chronic) hPMSC administration and the underlying mechanistic basis of stem cells in acute protection. Our current study represents the first step in such investigations and was accomplished primarily to determine whether and to what extent hPMSCs (when administered upon reperfusion) could acutely protect the brain in the murine MCAO model.

Despite its promise, SCT has nonetheless encountered several noteworthy considerations regarding their clinical application. When stem cells are administered intravenously (IV), only a very small fraction (<1%) actually penetrate the brain by 24 h [69], [70], [71]. In this study, the only compartments we have studied stem cells within are the peritoneum, blood and brain (**Fig. S6**). We monitored IP-injected hPMSCs in the circulation and found a maximal number of cells appearing in the vascular compartment at 6 h following administration; this corresponded to only <1% of injected cells (5000 cells in the 2.5 ml of blood/5 × 10^5^ cells given) (**Fig. S6b**). Although we could not identify a final destination of injected hPMSCs, the spleen and lungs are possible additional end points [70,72].

An important limitation of stem cell therapy has been IV administration, which can too often trigger intravascular coagulation and risks of injury or death [73], [74], [75]. In our hands, only 12% of ‘normal’ mice survived IV injection of hPMSC (*n* = 17) *even without receiving MCAO*. By comparison, our pilot studies showed that 87% of MCAO-stressed mice *survived* after IP injection of hPMSCs (*n* = 15), nearly identical to the survival rate in non-hPMSC treated MCAO mice (86%; 18 mice of 21) (**Table S2**). Therefore, we abandoned IV administration of hPMSCs early on in favor of IP delivery. Remarkably, we found that IP administration of 5 × 10^5^ hPMSCs at the beginning of reperfusion provided extremely potent and highly significant protection against tissue injury in the MCAO model of stroke (Fig. 1**a**). To our knowledge, our model for the first time shows that unlike IV administration, *intraperitoneal*-hPMSC administration is also highly effective, safe and well-tolerated as a therapeutic approach in MCAO (**Table S2**). Most strikingly, and mechanistically, hPMSC-treated mice subjected to MCAO showed a significant preservation of CBF compared to MCAO-treated mice at 24 h **(**Fig. 1**b-d).** This pattern also found maintenance of normal blood flow to the ischemic brain hemisphere when hPMSCs were administered following MCAO, a result that was also associated with significantly maintenance of surviving neurons (shown by Nissl staining) (Fig. 1**f-i**) and protection of neurological function (Fig. 1**e**). Our observation that there was a severe CBF decrease in ipsilateral hemisphere of MCAO mice stands in contrast to some findings in the literature which reflect when blood flow measures were recorded. Several previous stroke studies have measured CBF immediately after occlusion (i.e. at the beginning of reperfusion) [76,77]. However, in our study we performed Laser Speckle imaging of blood flow 24 h after reperfusion. The severe reduction in contralateral CBF of MCAO group in our study appears to relate to an ongoing and progressive vasoconstrictor provoked by ischemia/reperfusion injury. In support of this concept, we observed that cerebrovascular vasoconstriction and injury had not occurred at 4 h (**Fig. S1a),** that is to say loss of perfusion had not yet taken place by 4 h. Therefore, maintenance of blood flow using hPMSCs could still be effective at this point and later in the course of therapy. Future studies will be required to follow up on these findings to investigate how much later after reperfusion (i.e. beyond the 4 hr reperfusion ‘window’) hPMSCs may provide protection for stroke, currently the major limitation to administration of t-PA in stroke patients.

We have also obtained several lines of evidence indicating that hPMSCs protection against MCAO injury may be EV-dependent. First, we observed elimination of protection against MCAO injury when hPMSCs were pre-treated with the cholesterol chelator MβCD (Fig. 3). This agrees with studies suggesting that stem cell derived EVs account for *paracrine* benefits of SCT in stroke [12,[51], [52], [53]]. By depleting membrane cholesterol and disrupting lipid rafts [22,23,25], MβCD inhibits formation and release of EVs. Consequently, cholesterol availability may be an important co-factor needed to generate hPMSC-EVs in stroke protection [55]. Consistent with this, our studies demonstrated for the first time that cholesterol/lipid *supplementation* of hPMSCs enhanced the protective efficacy of hPMSCs at least 500% in our MCAO model. We found that 5 × 10^5^ hPMSCs were needed to protect the brain against MCAO injury (Fig. 1), but 20% of this number of hPMSCs (1 × 10^5^; Fig. 4) was not protective. Interestingly, when this low dose of hPMSCs (1 × 10^5^; Fig. 4**)** had been supplemented with cholesterol/lipid and administered IP significantly protected against MCAO induced injury (Fig. 4), consistent with cholesterol/lipid-treated hPMSCs being more ‘potent’ (based on an efficacious ‘dose’). We do recognize that the exact mechanisms by which cholesterol supplementation of the hPMSCs affords protection in this model remain unclear but consistent with our data using MβCD-treated hPMSCs, our findings support a role for EVs formation. This topic remains the subject of intensive and ongoing investigations.

While hPMSCs are an abundant, non-immunogenic and ethically ‘neutral’ source of EVs, EVs purified from hPMSCs have not yet been tested clinically for their protective effects in SCT. Some studies have shown that IV injection of hPMSC-derived EVs may be safe and protective in animal models [78,79]. One complication of IV administration of hPMSCs (or hPMSCs-derived EVs) has been activation of coagulation pathways [74,75] triggered by PS exposure and TF binding to the surface of stem cells [75,80]. We also found a high rate of mortality when hPMSC-derived EVs were given intravenously (**Table S2**). Differences between our findings and those in the literature [78,79] could reflect different isolation methods and culture media, species or mouse strain receiving the EVs. With respect to EVs in therapy, we found that cholesterol/lipid treatment of hPMSCs significantly reduced PS on the EV surface (Fig. 5). Most importantly IV injection of PS negative-sorted EVs (2 × 10^6^) from cholesterol/lipid-treated hPMSCs was non-traumatic and again significantly protected the brain against MCAO stroke injury (Fig. 5). These findings are consistent with PS status playing a critical role in safety and beneficial effects offered by hPMSC-derived EVs. In terms of scale, 5 × 10^5^ cells administered to a 27–30 g mouse (adjusted for the mass of a 70 kg human) indicates that an equivalent human IP dose would approach 1 × 10^9^ cells. While feasible, it could be technically challenging to produce such large amounts of cells. However, if cholesterol/lipid supplementation were employed, it is possible that only 20% of this dose (~2 × 10^8^) might be needed (Fig. 4). Future therapies for stroke might benefit from the use of cholesterol/lipid treated hPMSCs or EVs from these cells as potent therapeutics to stabilize cerebral perfusion, BBB and functional recovery following stroke. Experiments to validate these early findings in human clinical trials are still required.

Although we have demonstrated that beneficial effects of hPMSC appear to be mediated by the release of EVs, several questions remain to be answered. We showed that inhibiting EV formation by hPMSCs (using MβCD) eliminated protection against MCAO injury. However, it has been difficult to track the distribution of EVs upon release from hPMSCs. Several studies have introduced methods for labeling and imaging EVs [81,82] which might provide a possible tool to study EVs trafficking *in vitro* and *in vivo* and may help to define EVs biodistribution, and its relationship to their therapeutic activity.

In our current study we show that hPMSCs/EVs prevent a severe drop in CBF 24 h after the ischemic insult (Figs. 1**&** 3). It is also unclear as which cellular/molecular mechanisms are affected by hPMSC/EVs treatment to produce this benefit. Several studies indicated that MSCs/EVs exert protective effects by stimulating production of vasodilators e.g., nitric oxide (NO) and prostaglandins which increase local CBF [12]. Besides vasodilators, MSCs/EVs may provide vasoprotection by supporting endothelial and smooth muscle functions in autoregulation and vascular homeostasis [12,83,84]. Although our data are entirely consistent with these models, further studies will be required to validate molecular and cellular mechanisms underlying hPMSCs benefits.

In summary, our study demonstrates that protective actions of hPMSCs administration are mediated by release of extracellular vesicles which favorably impact CBF restoration in the post-MCAO brain, to potentially provide a highly translatable therapy for human stroke.

## Declaration of Competing Interest

A patent application by authors J.S.A., Y.W., and M.B. “Protective effect of intraperitoneal injection of human placenta stem cells in stroke” has been submitted to the LSUHSC-Shreveport office of sponsored programs and technology transfer. Other authors declare no competing interests.

## Authors contributions

Conceptualization, M.B. and J.S.A; Methodology, M.B., S.G.C., C.J.B., J.S.A.; Validation, M.B.; Formal Analysis, M.B., L.A.W., and X.L.; Investigation, M.B., R.S.E., and J.W.Y; Resources, Y.W., X.L., K.Y.S., and J.S.A.; Writing-Original Draft, M.B. and J.S.A.; Writing-Review & Editing, K.Y.S., X.L., R.E.K., and A.M.; Supervision, J.S.A., Y.W., O.C., R.E.K., and A.M.; Funding Acquisition, R.E.K., A.M., and J.S.A.

## Data sharing statement

All datasets generated for this study are included in the manuscript and/or the supplementary materials.

## Patent applications

LSUHSC-S-2019–03 “Protective effect of intraperitoneal injection of human placenta stem cells in stroke”.

## Appendix Supplementary materials

Image, application 1

Image, application 2

Image, application 3

Image, application 4

Image, application 5

Image, application 6

Image, application 7
