# Supplementary material for: Human placental mesenchymal stem cells improve stroke outcomes via extracellular vesicles-mediated preservation of cerebral blood flow
Source: eBioMedicine. 2020 Dec 19;63:103161. doi: 10.1016/j.ebiom.2020.103161 (PMC7753936; doi:10.1016/j.ebiom.2020.103161)
Supplement: Supplementary file 1 [file mmc1.docx]

**Supplementary Materials**


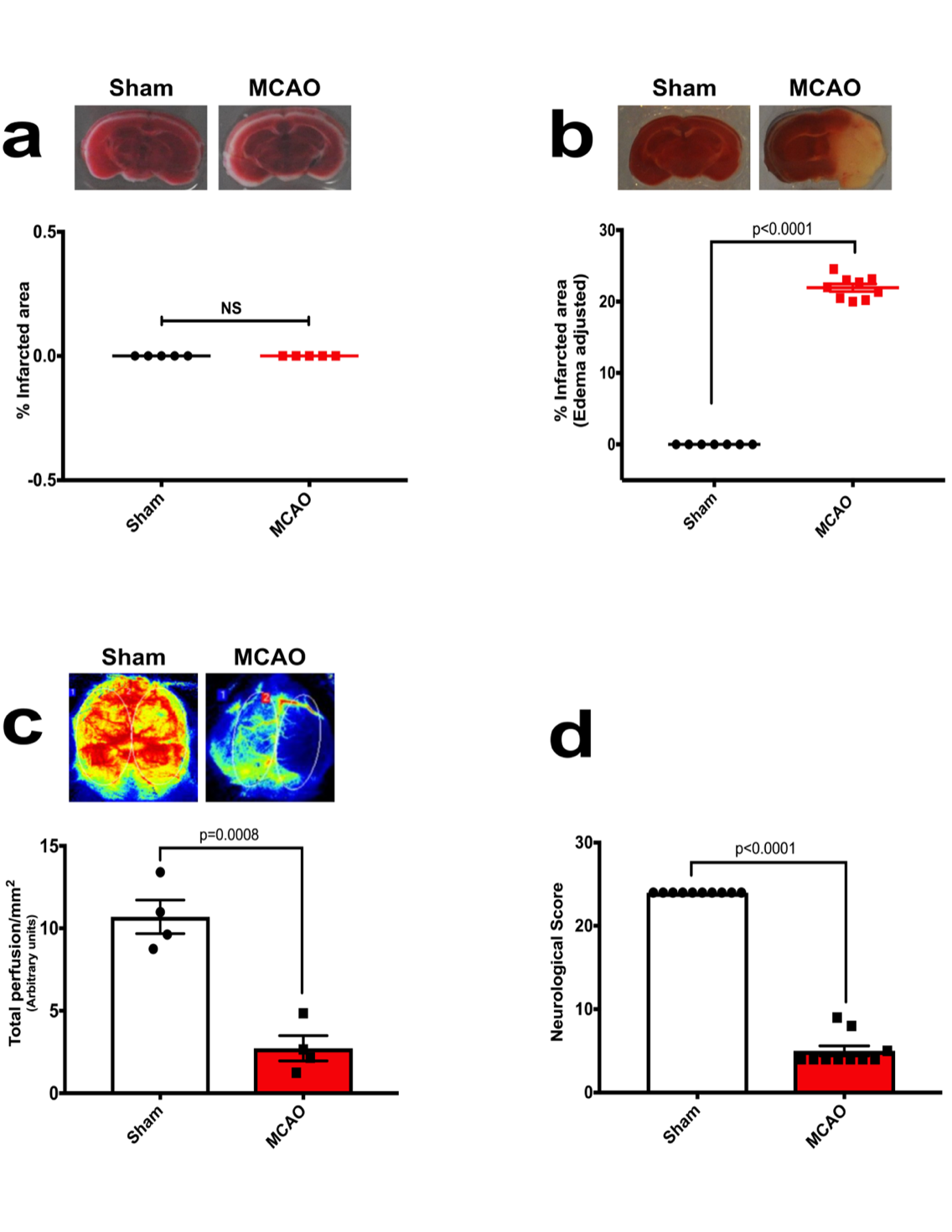


**Figure S1. MCAO model of Stroke.**

(a) There were no significant differences in infarction between MCAO (n=4) and sham group (n=4) at time point of 4 hours following reperfusion (*NS*, Student t-test analysis).

(**b**) Significant differences of infarcted area (TTC staining) in MCAO mice (n=10) versus sham group (n=7) was detected 24 hours after reperfusion using Student t-test analysis, *^****^P* < 0.0001.

(**c**) Significant differences of total perfusion into the brain (Laser Speckle Imaging) of MCAO mice (n=4) versus sham group (n=4) was detected 24 hours after reperfusion using Student *t*-test analysis, ^****^*P* = 0.0008.

(**d**) Significant differences of neurological scores in MCAO mice compared to sham group was detected 24 hours after reperfusion using Student *t*-test analysis, ^****^*P* < 0.0001.


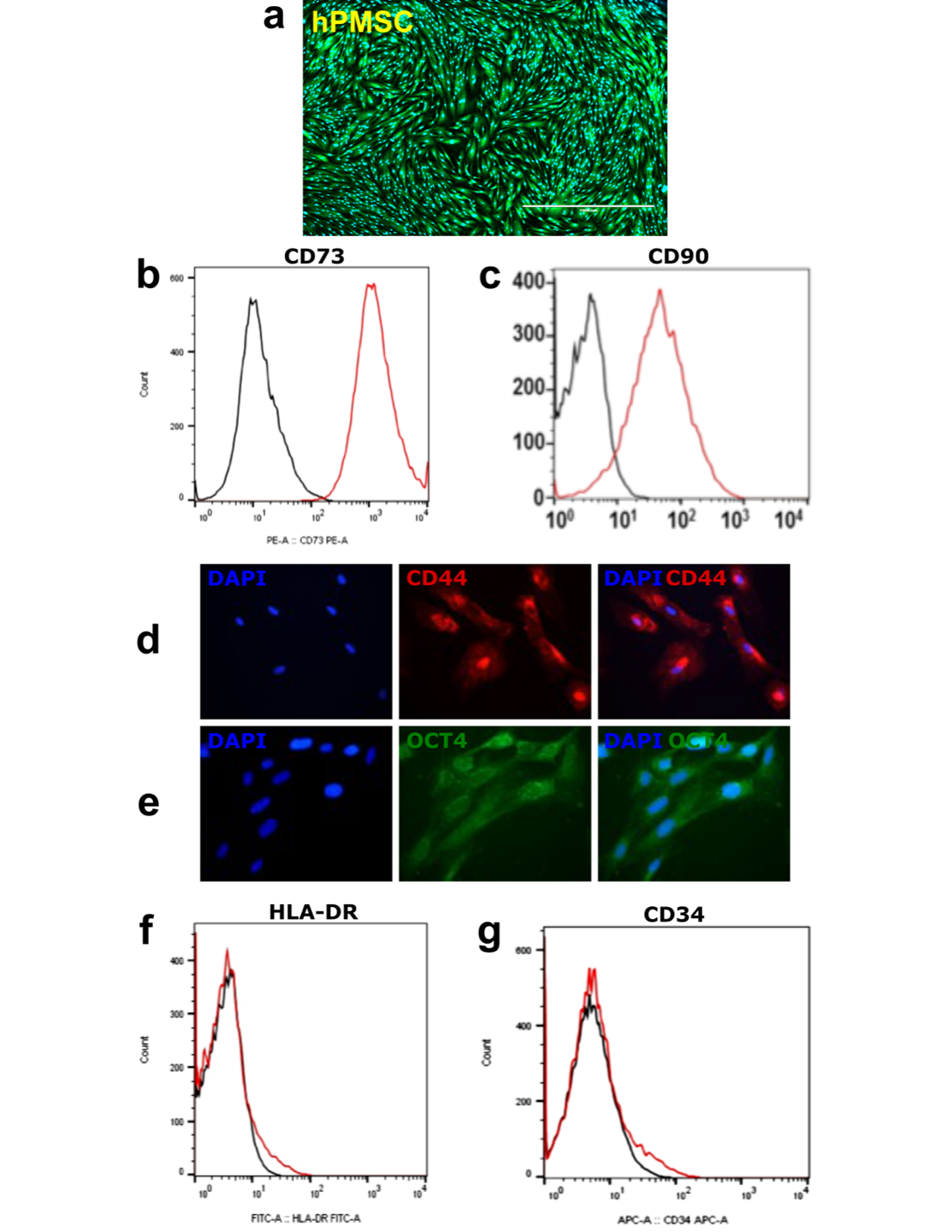


**Figure S2. Characteristics of hPMSC**.

(**a**) Calcein AM staining showed spindle shape of hPMSCs in culture. Scale bar, 100μm. Fluorescence-activated cell sorting (FACS) analysis detected the expression of CD73 (**b**) and CD90 (**c**) on hPMSC. Immunostaining of hPMSC was positive for markers of CD44 (**d**) and Oct3/4 (**e**). DAPI used for nuclear staining (**a**, **d** and e).

(**f** and **g**) FACS analysis showed negative expression of HLA-DR and CD34 markers on hPMSC.


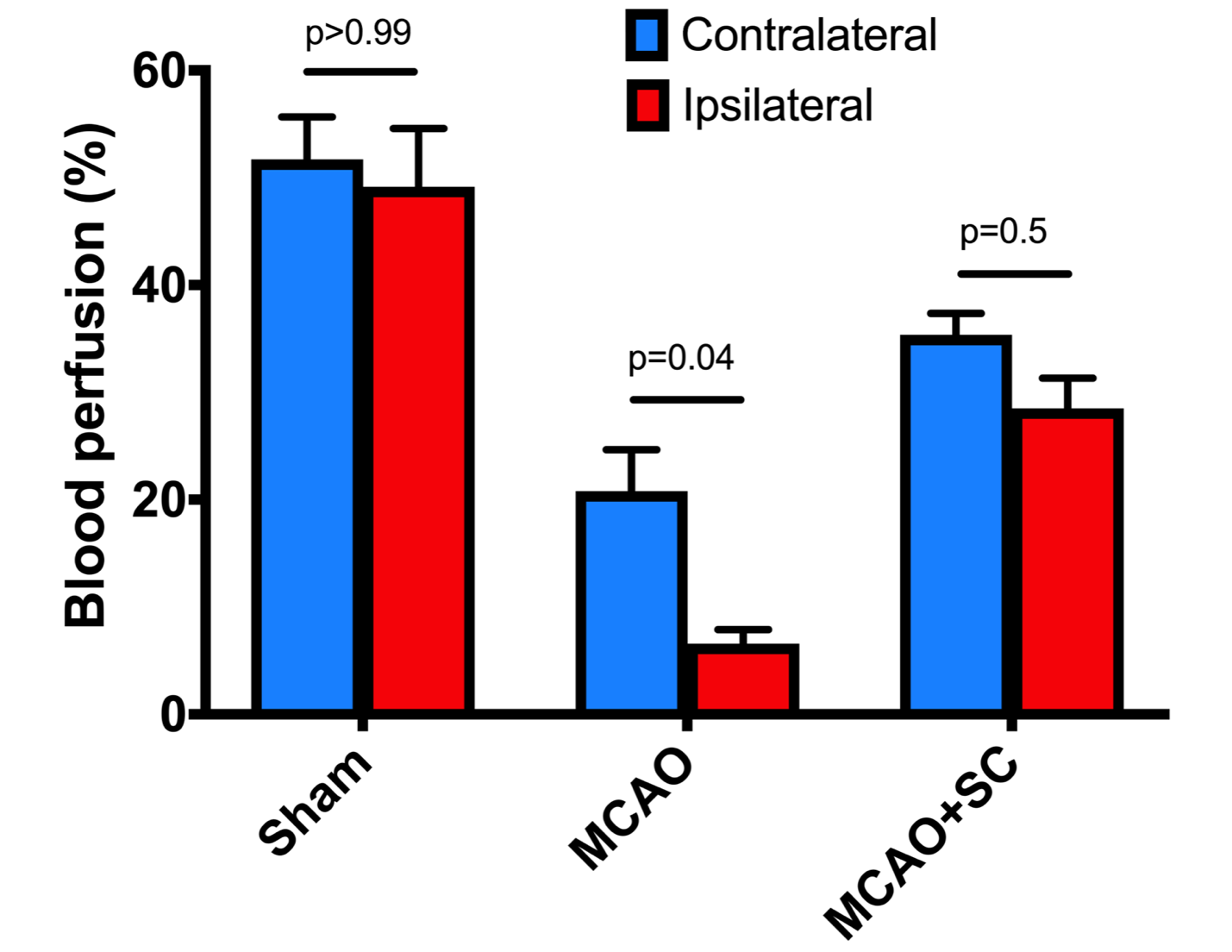


**Figure S3. Comparison of blood flow between contralateral and ipsilateral hemispheres.**

Laser Speckle imaging analysis showed no significant differences in blood perfusion of contralateral (51.71%) versus ipsilateral (49.17%) hemisphere of sham animals (NS; *p*>0.99; two-way ANOVA). Two-way ANOVA analysis revealed significant differences between contralateral perfusion (20%) compared to ipsilateral (6%) site of MCAO group (*p*=0.04). In comparison, there was no significant differences between contralateral (35%) and ipsilateral (29%) hemispheres of MCAO mice treated with hPMSC (NS; *p*=0.5).


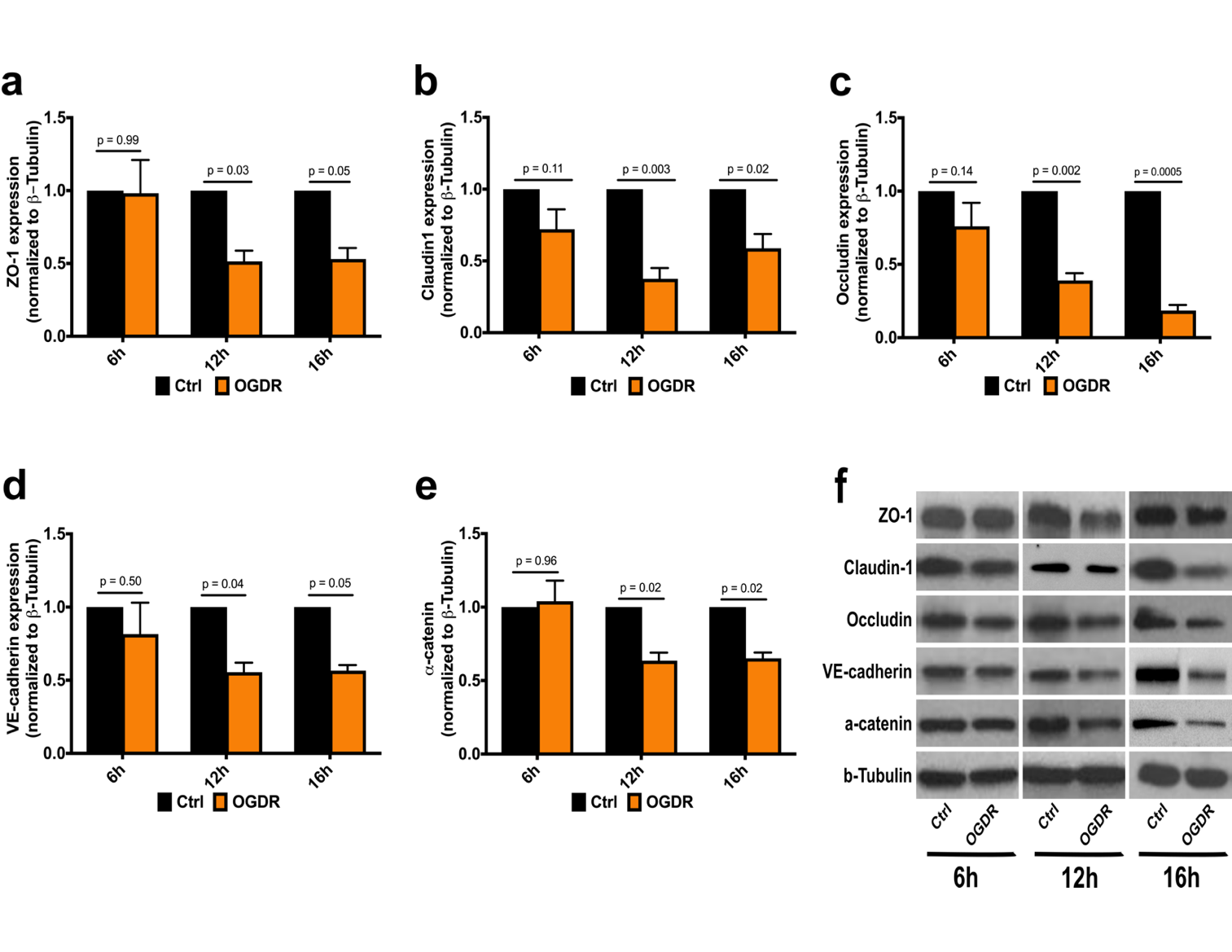


**Figure S4. Expression of tight/adherens junctional proteins under OGDR condition.**

Protein expression of tight junction proteins (ZO-1, claudin-1, occludin) and adherens junction proteins (VE-cadherin and α-catenin) of hCMEC-D3 monolayers under oxygen glucose deprivation reperfusion (OGDR) condition at different time points of 6, 12, and 16 hours was measured by western blot analysis.

(**a-e**) Statistical differences were determined by two-way ANOVA and Sidak’s multiple comparisons tests for comparisons of OGDR condition at 6, 12, and 12 time points. For quantification, expression of each protein normalized to protein expression of β-tubulin. Significant differences in protein expression of each protein under OGDR compared to normoxia at different time points as follow: (a) ZO-1 (6h; *p* = 0.99, 12h; *p* = 0.03, 16h; *p* = 0.05);

(**b**) claudin-1 (6h; *p* = 0.11, 12h; *p* = 0.003, 16h; *p* = 0.02);

(**c**) occludin (6h; *p* = 0.14, 12h; *p* = 0.002, 16h; *p* = 0.0.005);

(**d**) VE-cadherin (6h; *p*= 0.50, 12h; *p* = 0.04, 16h; *p* = 0.05);

(**e**) α-catenin (6h; *p* = 0.96, 12h; *p* = 0.02, 16h; *p* = 0.02).

(**f**) Representative western blots.

**
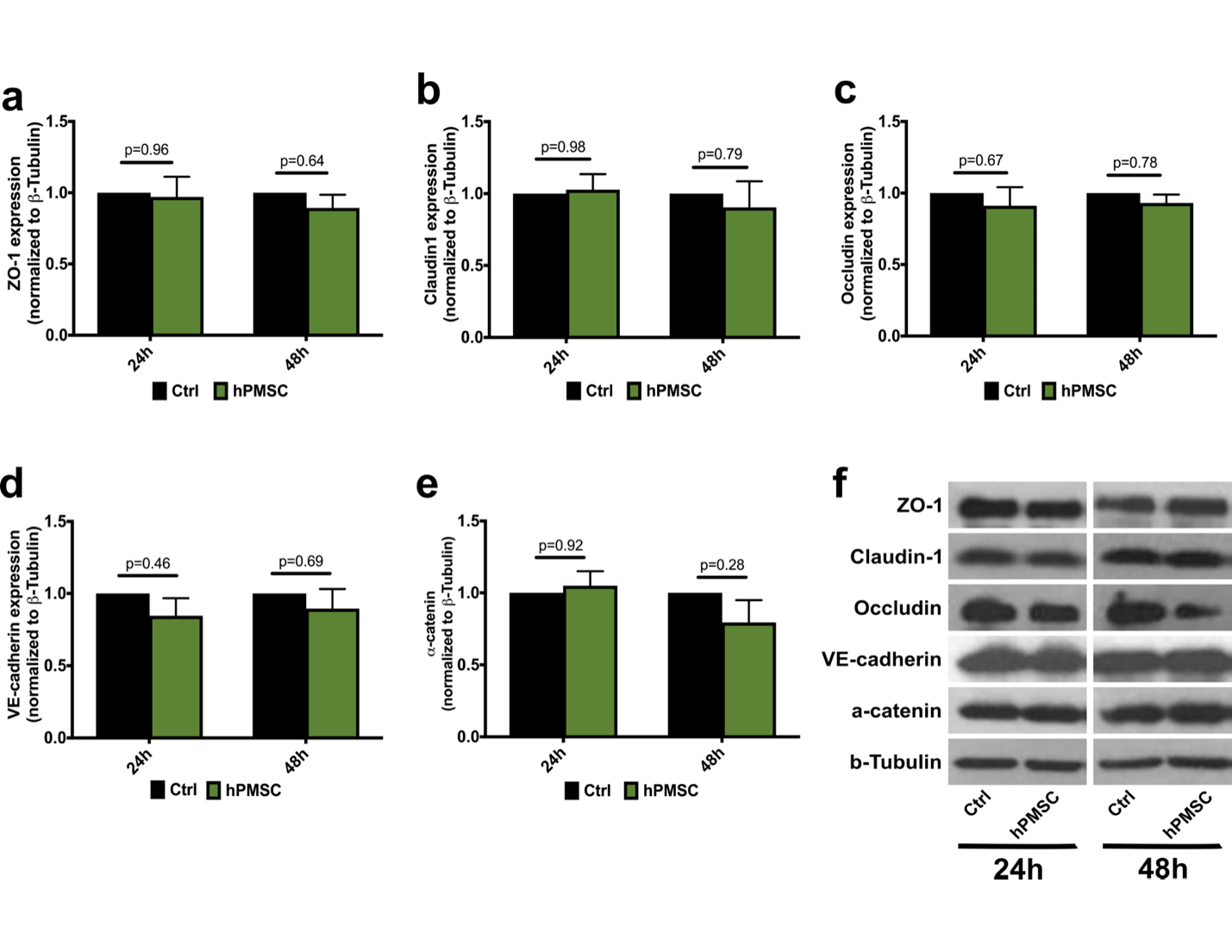
**

**Figure S5. Expression of tight/adherens junctional proteins of hCMEC-D3 monolayers co-cultured with hPMSCs under normoxia.**

(**a-f**) Protein expression of tight junctional proteins (ZO-1, claudin-1, occludin) and adherens junctional proteins (VE-cadherin and α-catenin) of hCMEC-D3 monolayers under normal (normoxia) condition when co-cultured (contact independently) with hPMSC for 24 hours and 48 hours compared to the correspondent control using western blot analysis. Statistical differences were measured by two-way ANOVA and Sidak’s multiple comparisons tests. For quantification, expression of each protein normalized to protein expression of β-tubulin. No significant differences were detected in protein expression of each protein in hCMEC-D3 monolayers ± co-cultured hPMSC (24, 48 hours) as shown here:

(**a**) ZO-1 (24h; *p* = 0.96, 48h; *p* = 0.64);

(**b**) claudin-1 (24h; *p* = 0.98, 48h; *p* = 0.78);

(**c**) occludin (24h; *p* = 0.67, 48h; *p* = 0.78);

(**d**) VE-cadherin (24h; *p* = 0.46, 48h; *p* = 0.69);

(**e**) α -catenin (24h; *p* = 0.92, 48h; *p* = 0.28).

(**f**) Representative western blots.


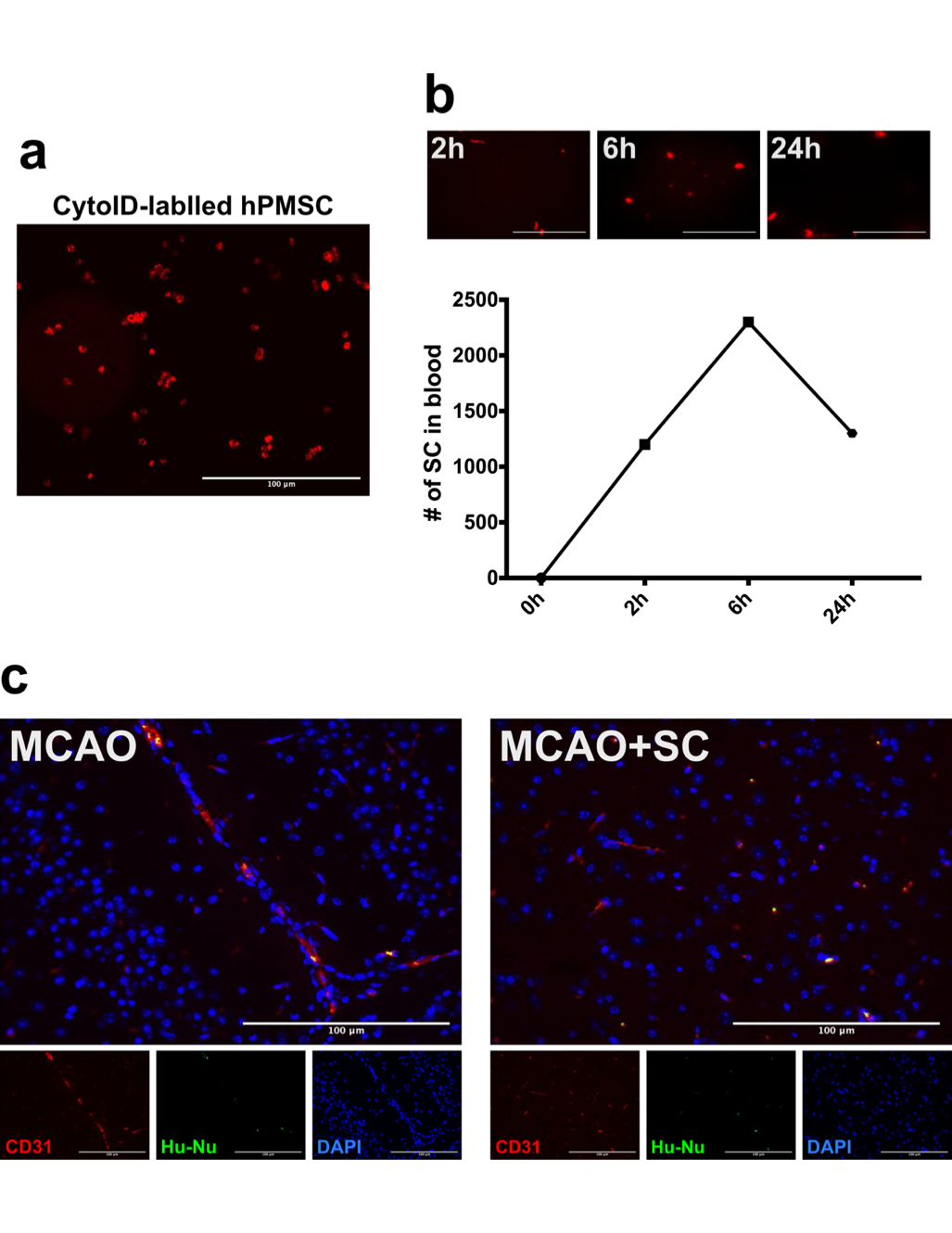


**Figure S6. hPMSCs do not integrate into the brain after ischemic injury.**

(**a**) To track intraperitoneally injected hPMSCs in the MCAO model, hPMSCs were labelled using CytoID red long-term cell tracer kit.

(**b**) Blood was collected at time points of 2, 6, and 24 hours after injection, and red-labelled cells were counted by a Nikon video imaging system Eclipse E600FN; using a 20X objective lens. Scale bars, 100μm.

(**c**) IF staining of hPMSC with anti-human nuclear (Hu-Nu; green) antibody and mouse vascular endothelial cells with anti-mouse CD31 antibody (red); DAPI was used to stain the nuclear of the cells. There was no hPMSC localized in the brain (no green signal was detected). Scale bars, 100μm.


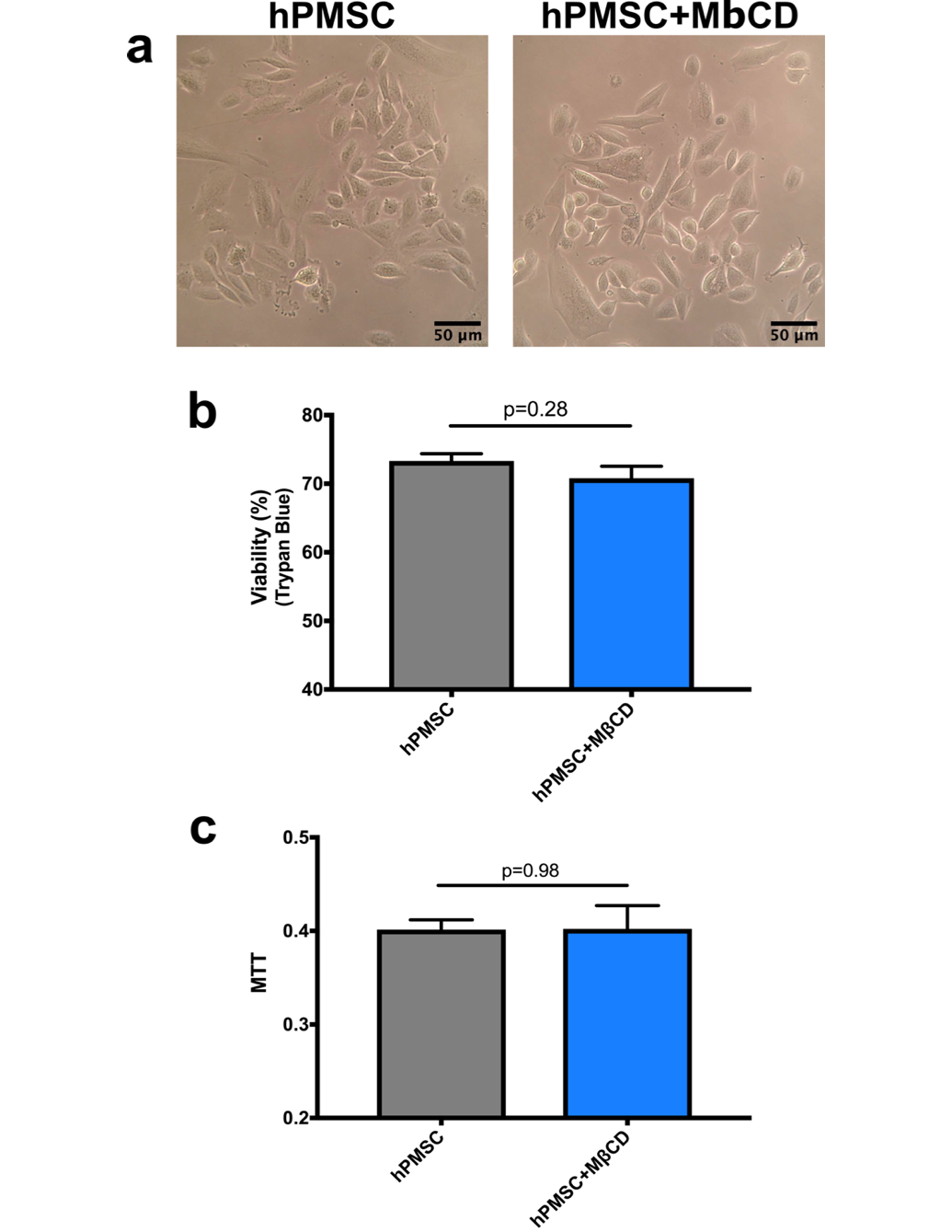


**Figure S7. Evaluation of MβCD-treated hPMSCs viability.**

**(a)** Binocular microscope imaging (20X) did not show any morphological changes between hPMSCs (left panel) and MβCD-treated hPMSCs (right panel). Scale bars, 50μm.

**(b)** Trypan blue test of viability. No significant differences were detected between %viability of hPMSCs and MβCD-treated hPMSCs using Student *t*-test analysis (NS; *p*=0.28).

**(c)** MTT assay did not show any significant differences between hPMSCs and MβCD-treated hPMSCs (NS; *p*=0.98, Student *t*-test analysis).
