# Supplementary material for: Human placental mesenchymal stem cells improve stroke outcomes via extracellular vesicles-mediated preservation of cerebral blood flow
Source: eBioMedicine. 2020 Dec 19;63:103161. doi: 10.1016/j.ebiom.2020.103161 (PMC7753936; doi:10.1016/j.ebiom.2020.103161)
Supplement: Supplementary file 2 [file mmc2.docx]

|  | 0 | 1 | 2 | 3 |
| --- | --- | --- | --- | --- |
| Spontaneous activity (5 min) | No movement | Slight movement | Touches 1 or 2 sides of cage | Touches 3 or 4 sides of cage |
| Symmetry of movement | Left side: no movement | Left side: slight movement | Left side: moves slowly | Both side: move symmetrically |
| Response to vibrissae touch |  | No response on left side | Weak response on left side | Symmetrical response on left side |
| Floor walking | No walking | Walks in circles only | Curvilinear path | Straight path |
| Beam walking | Falls off of beam | Hugs beam | Stands on beam | Walks on beam |
| Symmetry of forelimbs (outstretching while held by tail) | Left side: no movement, no outreaching | Left side: slight movement to outreach | Left side: moves and outreaches less than right side | Symmetrical outreach |
| Climbing wall of wire cage |  | Fails to climb | Left side is weak | Normal climbing |
| Reaction to touch on either side of trunk |  | No response on left side | Weak response on left side | Symmetrical response |

**Table S1. Neurological behavior parameters.**
