# Supplementary material for: Human placental mesenchymal stem cells improve stroke outcomes via extracellular vesicles-mediated preservation of cerebral blood flow
Source: eBioMedicine. 2020 Dec 19;63:103161. doi: 10.1016/j.ebiom.2020.103161 (PMC7753936; doi:10.1016/j.ebiom.2020.103161)
Supplement: Supplementary file 3 [file mmc3.docx]

| **Treatment** | **Injection route** | **Survival (%)** |
| --- | --- | --- |
| MCAO | _ | 86 |
| hPMSCs | i.v. | ~12 |
| MCAO+hPMSCs | i.p. | ~87 |
| MCAO+IV-PS^+^ EVs | i.v. | 15 |
| MCAO+IV- PS^-^ EVs | i.v. | 80 |

**Table S2. Survival rate of mice after IP or IV injection of hPMSCs or hPMSCs-derived EVs.**

Row 1: 86% of MCAO mice (non-hPMSCs treated) survived (n=21).

Row 2: 12% of normal mice (non-MCAO) survived IV injection of hPMSCs (n=17).

Row 3: 87% of MCAO mice survived IP injection of hPMSC (n-15).

Row 4: 15% of MCAO mice survived IV injection of PS-positive EV collected from untreated-hPMSC (n=7).

Row 5: 80% of MCAO mice survived IV injection of PS-negative EV collected from cholesterol-treated hPMSC (n=10).
