# Supplementary material for: Human placental mesenchymal stem cells improve stroke outcomes via extracellular vesicles-mediated preservation of cerebral blood flow
Source: eBioMedicine. 2020 Dec 19;63:103161. doi: 10.1016/j.ebiom.2020.103161 (PMC7753936; doi:10.1016/j.ebiom.2020.103161)
Supplement: Supplementary file 4 [file mmc4.docx]

**Original Western Blots**

Western Blots for supplementary figure 5: Expression of junctional proteins in hCMEC-D3 monolayers co-cultured with hPMSC under normoxia after **24 h**.
