# Supplementary material for: Human placental mesenchymal stem cells improve stroke outcomes via extracellular vesicles-mediated preservation of cerebral blood flow
Source: eBioMedicine. 2020 Dec 19;63:103161. doi: 10.1016/j.ebiom.2020.103161 (PMC7753936; doi:10.1016/j.ebiom.2020.103161)
Supplement: Supplementary file 6 [file mmc6.docx]

Western Blots for supplementary figure 4. Expression of tight/adherens junctional proteins under OGDR condition at 6, 12, and 16 hours.
