# Supplementary material for: Human placental mesenchymal stem cells improve stroke outcomes via extracellular vesicles-mediated preservation of cerebral blood flow
Source: eBioMedicine. 2020 Dec 19;63:103161. doi: 10.1016/j.ebiom.2020.103161 (PMC7753936; doi:10.1016/j.ebiom.2020.103161)
Supplement: Supplementary file 7 [file mmc7.docx]

Western blots for figure 2: Expression of tight/adherens junction proteins under OGDR condition with/without hPMSC treatement.
